# Evaluation of Pyrazolone-Based Hydrazones as Potential Therapeutic Agents Against Glioblastoma

**DOI:** 10.3390/ph19091335

**Published:** 2026-08-24

**Authors:** Giorgio Cameli, Alessia Piergentili, Eleonora Spinozzi, Alessia Tombesi, Riccardo Petrelli, Loredana Cappellacci, Maria Beatrice Morelli

**Affiliations:** 1 Immunopathology and Molecular Medicine Unit, School of Pharmacy, University of Camerino, Via Madonna delle Carceri 9, 62032 Camerino, Italy; giorgio.cameli@unicam.it; 2 Chemistry Interdisciplinary Project (ChIP) Research Center, School of Pharmacy, University of Camerino, Via Madonna delle Carceri 9, 62032 Camerino, Italy; alessia.piergentili@unicam.it (A.P.); eleonora.spinozzi@unicam.it (E.S.); alessia.tombesi@unicam.it (A.T.); loredana.cappellacci@unicam.it (L.C.)

**Keywords:** glioblastoma, pyrazolone-based hydrazones, antitumor activity

## Abstract

**Background**: Glioblastoma (GBM) is the most aggressive subtype of malignant glioma. Current therapeutic options remain limited, highlighting the need for the development of novel compounds capable of improving the efficacy of standard treatments. This study aimed to evaluate the biological activity of a series of pyrazolone-based hydrazone compounds (TPPs) in vitro GBM cell lines. **Methods**: The eight TPPs were synthesized by a nucleophilic addition reaction of different substituted hydrazines with 1-(5-hydroxy-3-methyl-1-phenyl-1*H*-pyrazol-4-yl)-2-phenylethan-1-one and tested on two human GBM cell lines, T98 and U87. The cytotoxic effects were evaluated via MTT assay. The most active compound was further investigated at IC_50_ and IC_25_ concentrations to evaluate mechanisms of cellular damage, including reactive oxygen species (ROS) production and mitochondrial membrane potential (ΔΨ_m_) changes. Additional assays included colony formation, cell cycle analysis, and evaluation of DNA damage and apoptosis markers. **Results**: TPP25 exhibited the highest activity with IC_50_ values of 11.01 μM (95% CI: 10.42 to 11.64) and 13.12 μM (95% CI: 10.23 to 16.87) on T98 and U87 lines, respectively. Treatment induced early ROS production and mitochondrial depolarization, along with a significant reduction in colony formation. Cell cycle analysis revealed accumulation in the sub-G0 phase, consistent with increased cell death, supported by propidium iodide uptake. Furthermore, the results suggest the involvement of an apoptotic-like mechanism as supported by Annexin V positivity, γ-H2AX upregulation and transient caspase-3 activation. **Conclusions**: TPP25 demonstrates significant in vitro cytotoxicity, likely driven by a pro-apoptotic mechanism. This profile positions it as a potential lead compound for further preclinical evaluation, supporting its future transition into in vivo GBM models.

## 1. Introduction

Glioma treatment remains one of the major challenges in oncology due to its highly aggressive nature and resistance to conventional therapies. Among gliomas, glioblastoma (GBM), classified as a grade IV tumor, represents the most common and aggressive form of primary brain tumors. In addition to its aggressiveness, GBM is characterized by a high degree of inter- and intratumoral heterogeneity, which significantly contributes to therapeutic resistance and disease progression [1]. According to the Central Brain Tumor Registry of the United States, GBM is the most prevalent and malignant subtype of glioma [2]. Apart from rare cases associated with genetic predisposition, such as hereditary retinoblastoma and syndromes including Cowden, Turcot, Lynch, Li-Fraumeni, and Maffucci, and exposure to high levels of ionizing radiation, no well-established risk factors for GBM development have been identified [3]. At the molecular level, GBM malignancy and therapeutic response are governed by key genetic and epigenetic biomarkers that drive the intrinsic heterogeneity. *Isocitrate dehydrogenase 1* or *2* (*IDH1/2*) mutations are exceedingly rare in primary GBM, with fewer than 10% of cases harboring these alterations; thus, the vast majority of patients present with aggressive *IDH*-wild-type tumors associated with dismal survival rates [4]. Concurrently, epigenetic silencing via *O^6^-methylguanine-DNA methyltransferase* (*MGMT*) promoter hypermethylation occurs in roughly 40–45% of patients, serving as the premier clinical marker for temozolomide (TMZ) chemosensitivity [4]. Conversely, the remaining 55–60% of patients with unmethylated *MGMT* promoters exhibit rapid, intrinsic resistance to alkylating agents due to efficient DNA repair.

Despite advances in multimodal approaches, including surgery, radiotherapy, and chemotherapy, the prognosis for GBM patients remains poor, with a median survival of less than two years. Therefore, the development of novel active compounds is urgently needed to enhance the efficacy of current therapeutic strategies for GBM.

Compounds containing pyrazolone and hydrazone moieties feature versatile functional groups with a wide range of biological properties. Pyrazolones represent one of the oldest classes of synthetic pharmaceutical compounds [5], and their derivatives play a highly significant role in the synthesis of numerous drugs [6]. Kulkarni et al. [7] reported the synthesis of a series of coumarin–pyrazolone hybrid compounds that were evaluated in vitro on different human tumor cell lines, among which glioma cell lines, and demonstrated moderate cell-proliferation inhibition.

On the other hand, in several works, hydrazones have been evaluated for their antioxidant, anti-inflammatory, antibacterial [8], analgesic [9], antiparasitic [10], antitubercular [11], anti-HIV [12], and antitumor activity [13,14]. Concurrently, recent developments have highlighted the potential of specialized hydrazone derivatives in targeting GBM cells [15], while related pyrazolone-based hydrazone ligands have demonstrated robust in vitro cytotoxicity in other solid tumor models [13]. Therefore, due to their high versatility, these molecules are considered valuable in medicinal chemistry.

**Figure 1:**
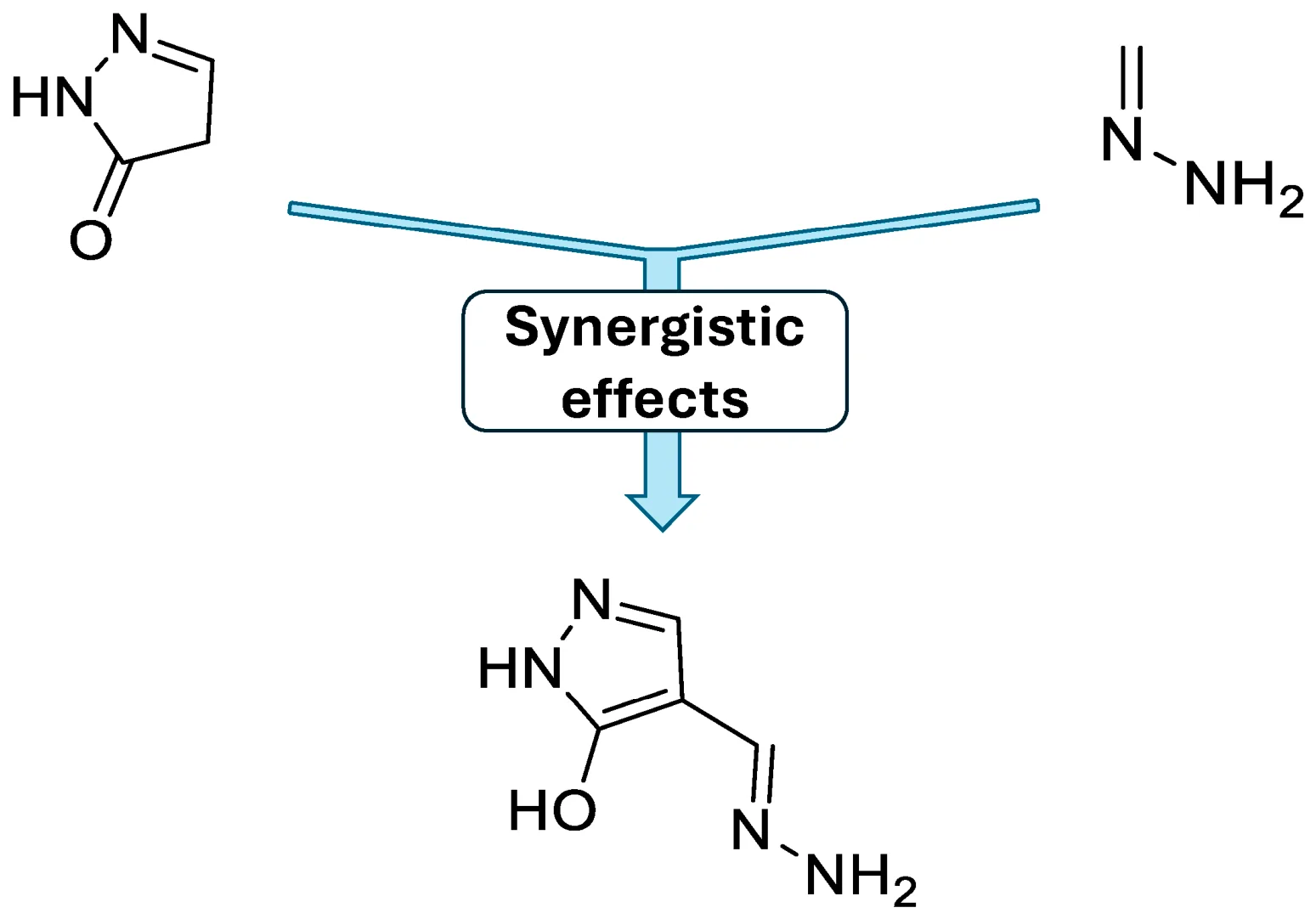
Design of pyrazolone-based hydrazones.

Based on the reasons outlined above, the objective of this work was to combine the hydrazone scaffold, which has a wide spectrum of antitumor activity, and the pyrazolone scaffold to synthesize a novel series of pyrazolone-based hydrazones (TPPs), exploiting the potential synergistic pharmacological effects of these two functional groups (Figure 1). We hypothesized that merging the lipophilic hydrazone motif pyrazolone into a single chemical entity would yield an advantageous combination capable of optimizing plasma membrane permeation and intracellular accumulation. This specific arrangement was hypothesized to generate a unique cytotoxic profile, thereby bypassing traditional chemoresistance mechanisms. While both pharmacophores have been independently investigated, their specific uncoordinated combination remains completely unexplored in GBM models. The newly synthesized compounds were evaluated against human GBM cell lines T98 and U87.

## 2. Results

### 2.1. Synthesis of the Pyrazolone-Based Hydrazone Compounds

In this work, a series of hydrazones was synthesized from 1-(5-hydroxy-3-methyl-1-phenyl-1*H*-pyrazol-4-yl)-2-phenylethan-1-one (HQ^Ph,Bn^) [10]. To this common backbone, hydrazines differently substituted on the aromatic ring were added by a nucleophilic addition reaction. Hydrazines were selected to obtain a series of hydrazones comprising at least two strong electron-withdrawing groups (TPP08 and TPP25), a medium electron-withdrawing group (TPP60), a weak electron-withdrawing group (TPP62), two heterocyclic substituents (TPP26 and TPP95), a medium electron-donating group (TPP96), and one without substituents (TPP55), with the aim to evaluate their cytotoxic activity on human GBM cell lines.

**Scheme 1:**
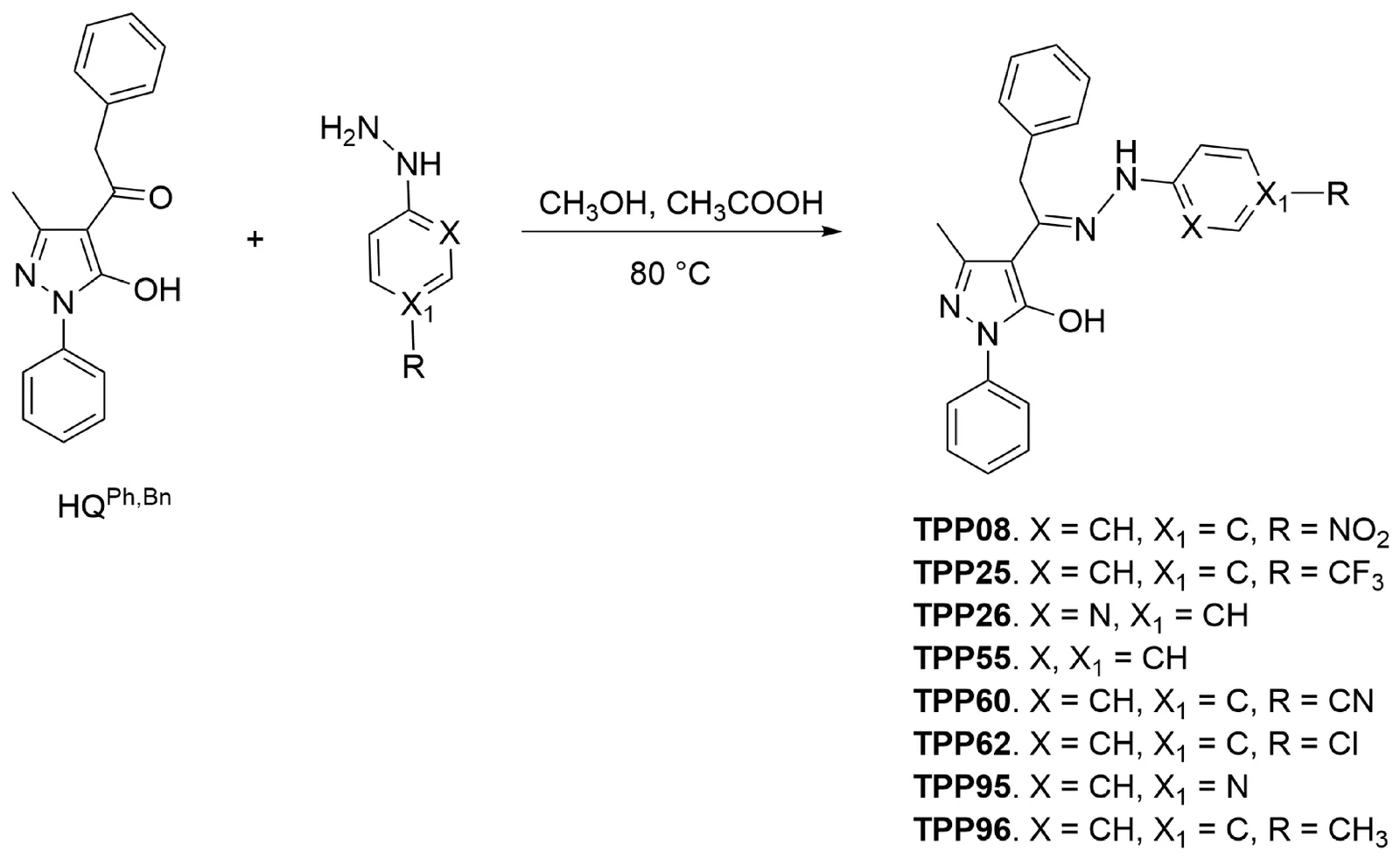
Synthesis of TPP series.

All the TPPs were obtained by the same synthetic process: To a solution of HQ^Ph,Bn^ in methanol, the respective hydrazine was added with glacial acetic acid as a catalyst. The reaction mixture was refluxed for 2–4 h at 80 °C. A precipitate slowly formed from the hot solution. The mixture was stored at 4 °C overnight, after which the precipitate was filtered and dried (Scheme 1).

All the synthesized hydrazones with their yields are reported in Figure 2. Most compounds in this series are characterized by spectroscopic data consistent with the enol tautomer commonly representative for other pyrazolone-based hydrazones. In contrast, TPP62 and TPP96 exhibit a distinct structural feature, as their spectroscopic data are consistent with the keto/bis-NH tautomer. This structural assignment is supported by the absence of the enolic OH signal in the ^1^H NMR spectra (see Section 4.2 Scheme 2, Scheme 3, Scheme 4, Scheme 5, Scheme 6, Scheme 7, Scheme 8 and Scheme 9 and Figures S1–S16).

### 2.2. Cytotoxic Activity

The biological activity of the TPP series was evaluated in T98 and U87 GBM cell lines by assessing their effects on cell viability. The half-maximal inhibitory concentration (IC_50_) values were determined after 72 h of treatment using the 3-(4,5-dimethylthiazol-2-yl)-2,5-diphenyltetrazolium bromide (MTT) assay, which are reported in Table 1 and Figures S17 and S18.

TPP25, bearing the trifluoromethyl group (–CF_3_), was found to be the most active compound of the series with IC_50_ values of 11.01 and 13.12 μM on T98 and U87 lines, respectively. The high activity of TPP25 is attributed to the strong electron-withdrawing effect of the –CF_3_ group, which contributes to a more favorable electronic distribution on the aromatic ring. Moreover, this substituent enhances the hydrophobic properties of the molecule (cLogP value of 6.43), improving its ability to cross the lipid bilayer of GBM tumor cells. Furthermore, the –CF_3_ group, despite being bulky, has a more compact geometry than the nitro group (–NO_2_) in TPP08, which showed IC_50_ values above 50 μM, indicating lower cytotoxic efficacy.

The presence of medium electron-withdrawing groups, such as nitrile (–CN) in TPP60, afforded an IC_50_ value of 49.32 μM against the U87 line and above 50 μM against T98. Despite a cLogP value of 5.07, which indicates moderate lipophilicity, the weaker electron-withdrawing effect of the –CN group compared with –CF_3_ may result in a less favorable electronic profile, leading to reduced biological activity. Similarly, the presence of weak electron-withdrawing groups, such as chlorine (–Cl) in TPP62, resulted in an IC_50_ above 50 μM against T98 and a non-determinable IC_50_ against U87. Although TPP62 exhibits a relatively high cLogP value of 6.13, comparable to that of TPP25, its poor cytotoxic activity suggests that lipophilicity is not sufficient to ensure potency and that the electronic nature of the substituent is essential for determining the cytotoxic activity.

The behavior of TPP55, which lacks substituents on the aromatic ring, is interesting, with IC_50_ values of 28.97 and 23.07 μM on T98 and U87 cell lines, respectively. However, in this case, the IC_50_ values are also higher than those of TPP25, as it lacks hydrophobic contributions (cLogP of 5.25) and additional favorable electronic distribution.

Heterocyclic derivatives TPP26 and TPP95, which have a nitrogen atom on the aromatic ring in positions 2 and 4, respectively, failed to yield a standard IC_50_ value within the tested concentration range, rendering both compounds unpromising. In particular, the replacement of a carbon with a nitrogen atom reduces the lipophilicity of the molecule with a cLogP of 4.29, which may impair the diffusion through the lipid bilayer of GBM tumor cells. However, they exhibited distinct pharmacological profiles: while TPP95 showed a complete lack of cytotoxicity up to 50 μM, TPP26 induced a clear reduction in cell viability (reaching approximately 33–60%) but displayed an anomalous, non-monotonic concentration–response relationship that could not be fitted to a standard sigmoidal model (Figures S17 and S18). Even TPP96, with a methyl group (–CH_3_), a medium electron-donating group, showed IC_50_ values greater than 50 μM. This outcome may be due to the reduction in hydrophobic properties (showing a cLogP of 5.75) and unfavorable electronic distribution.

**Figure 3:**
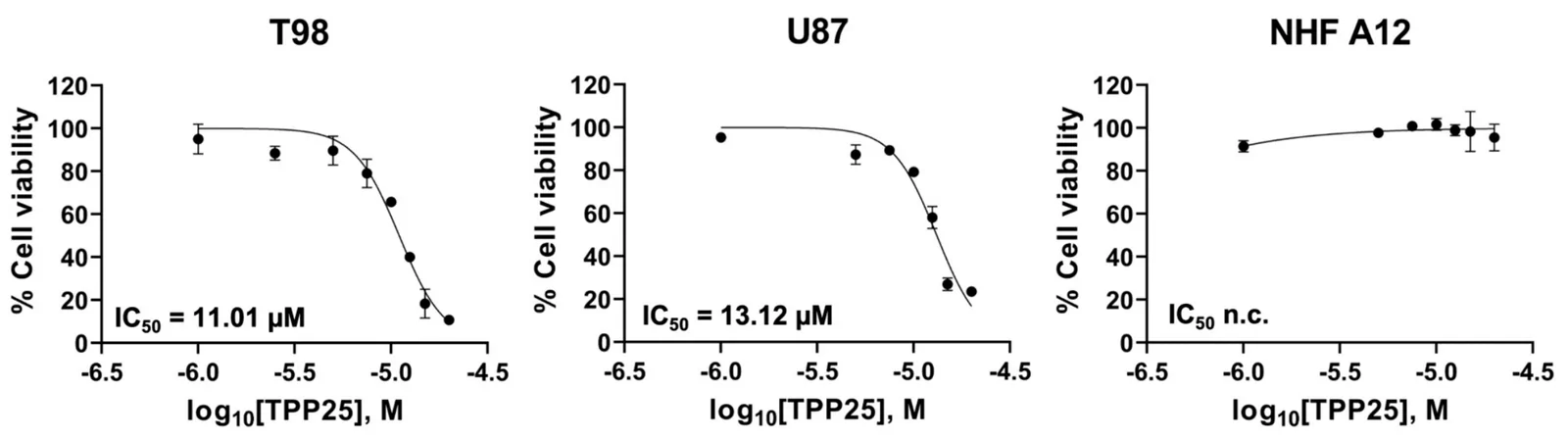
Cytotoxicity of TPP25 in T98, U87, and NHF A12 cell lines was determined by MTT assay. Cell viability was evaluated after 72 h post-treatment with TPP25 at various concentrations ranging up to 20 μM. Vehicle control cells were treated with DMSO at a final concentration < 0.05% (*v*/*v*), showing no statistically significant differences compared to completely untreated cells. Concentrations on the x-axis are expressed as the base-10 logarithm of the molar concentration (log_10_[TPP25], M). IC_50_ represents the drug concentration (μM) required to inhibit 50% of cell growth; n.c. (not cytotoxic) indicates a lack of cytotoxicity within the tested concentration range. Data shown are expressed as mean ± Standard Error of the Mean (SEM) of three separate experiments.

In conclusion, the combination of electronic and steric effects plays a key role in the antitumor activity of the synthesized TPPs against GBM. Among the tested compounds, TPP25, characterized by the presence of the strong electron-withdrawing group –CF_3_, stands out as the most promising candidate, exhibiting the lowest IC_50_ value and, consequently, the highest cytotoxic activity (Table 1, Figure 3). An additional relevant finding was obtained by evaluating the effect of TPP25 on the viability of primary NHF A12 cells (non-tumoral human fibroblasts), used as a model of non-tumoral cells to determine its selectivity profile. Notably, the compound exhibited no appreciable cytotoxicity against the non-malignant line, with cell viability remaining unaffected even at the highest tested concentration, resulting in an IC_50_ value of >20 μM (Figure 3). To quantify this therapeutic window, the Selectivity Index (SI) was calculated for each cell line. TPP25 demonstrated a clear, promising selectivity toward the malignant phenotypes, displaying a formal SI of >1.8 for T98 cells and >1.5 for U87 cells. While these results suggest a favorable in vitro selectivity window, further extensive evaluation across a broader panel of non-tumoral models, including normal human astrocytes, is required to comprehensively establish the global safety and biocompatibility profile of this derivative.

#### 2.2.1. Evaluation of Compound TPP25 on Human GBM Cell Growth

**Figure 4:**
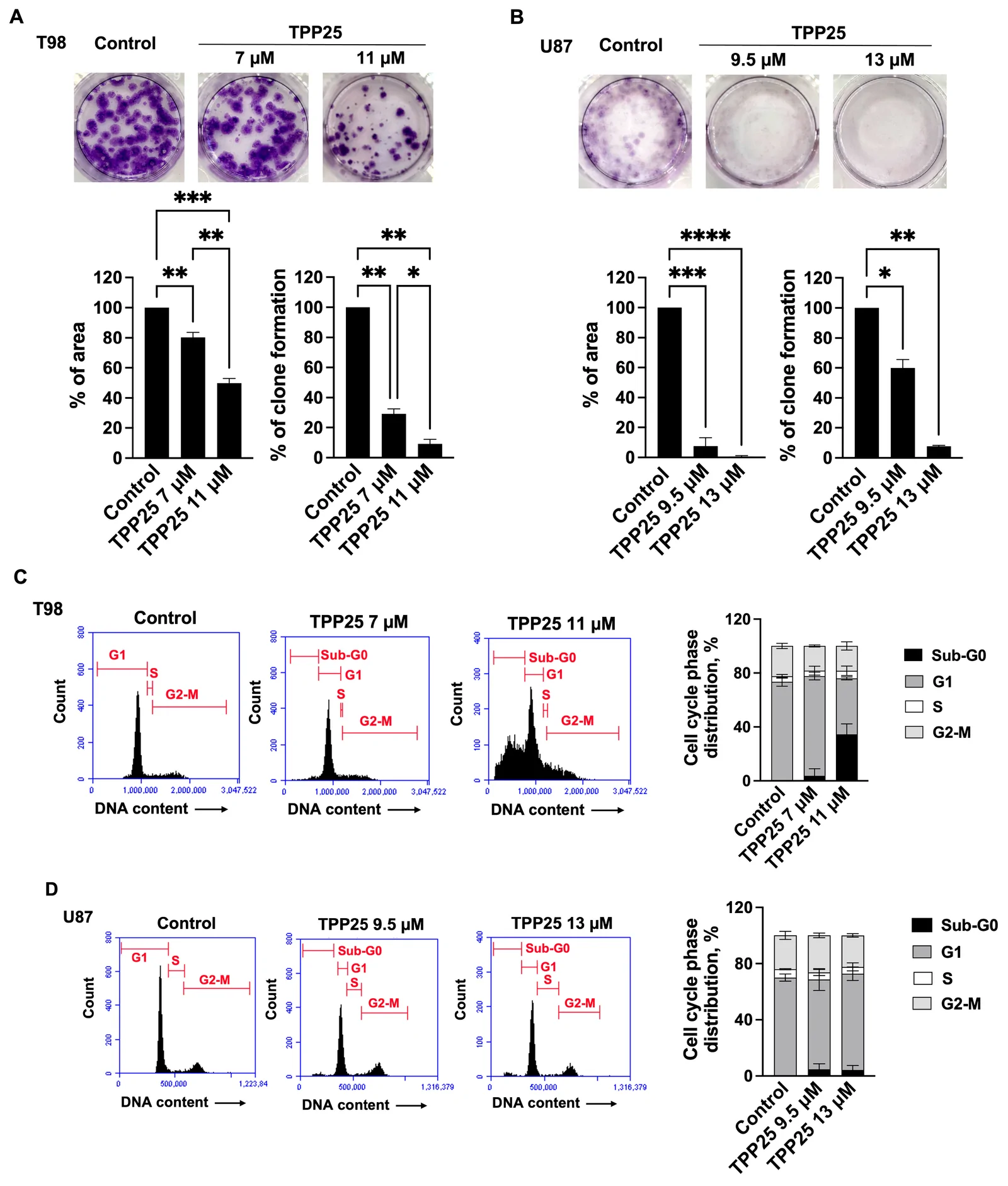
Effect of compound TPP25 on colony formation and cell cycle distribution in GBM cell lines. (**A**,**B**) Representative images of the clonogenic survival assay in (**A**) T98 and (**B**) U87 cells treated for 14 days with vehicle (control) or specific IC_25_ and IC_50_ doses of TPP25, followed by crystal violet staining (upper panel). Quantitative analysis showing the percentage of area occupied by colonies and the percentage of clone formation in both cell lines. Data are expressed as the mean ± Standard Error of the Mean (SEM) of three independent experiments. Statistical significance was determined using a one-way ANOVA followed by Tukey’s multiple comparison test to compare all treatment groups with each other. * *p* < 0.05, ** *p* < 0.01, *** *p* < 0.001, and **** *p* < 0.0001. (**C**,**D**) Representative flow cytometric histograms profiling the cell cycle distribution based on DNA content analysis in (**C**) T98 and (**D**) U87 cells after 72 h of treatment with the indicated concentrations of TPP25 (left panel). Bar graphs quantifying the percentage distribution of cells across the different phases of the cell cycle for (**C**) T98 and (**D**) U87 cells (right panel). Data are expressed as the mean ± Standard Error of the Mean (SEM) of three independent experiments.

Based on the initial cytotoxicity profiles, we further investigated the biological activity of TPP25 using both a sublethal concentration (IC_25_) and a fully cytotoxic dose (IC_50_) corresponding to the compound concentrations that reduce cell viability to 75% and 50% of the control, respectively. This dual-dose approach was chosen from a biological standpoint to capture the early, sublethal molecular events and signaling pathways triggered at the lower concentration before widespread and irreversible cell death pathways take over at the higher IC_50_ dose. First, the long-term proliferative capacity of the GBM cells was evaluated using a colony formation assay. Cells were treated for 14 days with the specific IC_25_ and IC_50_ doses of TPP25, followed by crystal violet staining. As shown in Figure 4A,B, treatments significantly suppressed colony formation in a concentration-dependent manner, with a more pronounced effect observed in the U87 line than in the T98 line. This suppression was validated by the quantitative analysis of both the percentage of area occupied by the colonies and the percentage of clone formation. Statistical analysis supports the notion that inhibitory effects were highly significant across all treated groups compared to the untreated control for both lines (*p* < 0.05), while a significant difference between the two tested concentrations was observed exclusively for T98 cells (Figure 4A) (*p* < 0.05). Interestingly, for U87 cells treated at the IC_25_ dose, a discrepancy was observed between the percentage of colony formation (~60%) and the percentage of area (~7%). This indicates that while the compound allows the survival of several colony-initiating units, it severely impairs their subsequent proliferation, resulting in a large number of microscopic, poorly expanded colonies. Conversely, at the higher dose (corresponding to the IC_50_), the treatment exerts a much more severe inhibitory effect, drastically reducing both the total colony count and the covered surface area, thereby demonstrating a dose-dependent drop in cell survival and expansion capacity. Collectively, these findings demonstrate that compound TPP25 effectively inhibits the long-term survival and growth of GBM cells in vitro.

To elucidate the mechanisms underlying this growth inhibition, we subsequently analyzed cell cycle distribution. Figure 4C,D show that vehicle-treated control cells were predominantly in the G1 phase, whereas TPP25 treatment prompted a shift toward the sub-G0 phase. Specifically, after 72 h of treatment, GBM cells significantly accumulated in the sub-G0 phase compared to vehicle-treated cells. This increase in the sub-G0 population was accompanied by a concomitant decrease in the percentage of cells in the G1 phase, without significant alterations in the remaining phases of the cell cycle. Notably, this imbalance was highly pronounced in the T98 cell line, whereas the U87 line exhibited only a minimal sub-G0 population.

#### 2.2.2. Effect of TPP25 on Cell Death

**Figure 5:**
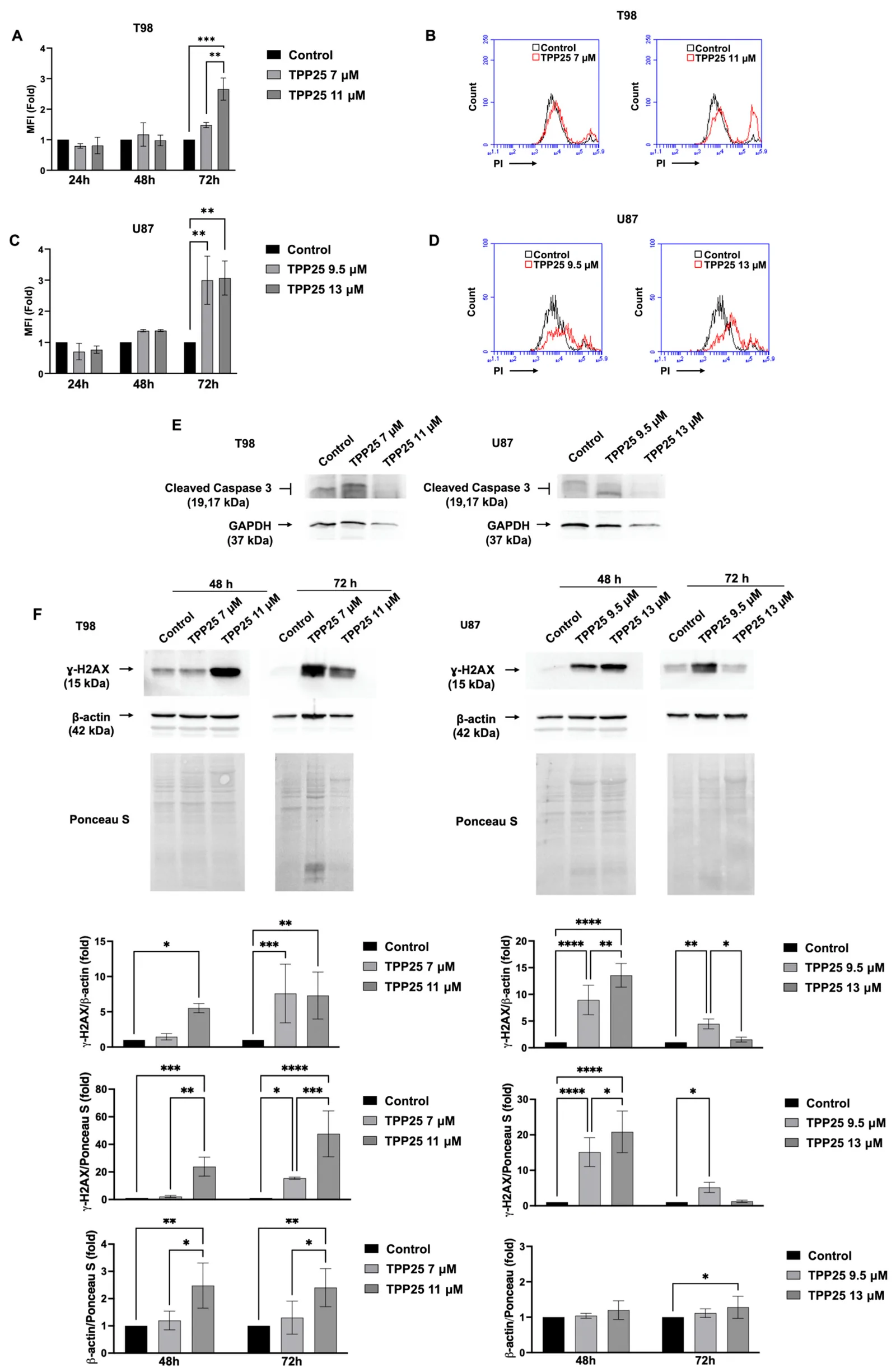
Evaluation of cell death in T98 and U87 GBM cell lines treated with TPP25. (**A**,**C**) Quantitative flow cytometric analysis of cell death using propidium iodide (PI) staining. Bar graphs represent the Mean Fluorescence Intensity (MFI) expressed as fold-change relative to control groups across three different time points for (**A**) T98 and (**C**) U87 cells. Data in the graphs are expressed as the mean ± Standard Error of the Mean (SEM) of three independent experiments. Two-way ANOVA with Tukey’s multiple comparison test was performed to analyze the effects of the two different treatment factors. ** *p* < 0.01; *** *p* < 0.001. (**B**,**D**) Representative flow cytometry overlay histograms at 72 h of treatment for (**B**) T98 and (**D**) U87 cells, comparing vehicle-treated control populations (black line) with TPP25-treated groups (red line) at the indicated doses. (**E**) Representative Western blot of Cleaved Caspase-3 and GAPDH expression after 72 h of treatment in T98 and U87 cells. (**F**) Western blot analysis of Phospho-H2AX (γ-H2AX) expression levels after 48 h and 72 h of treatment in T98 and U87 cells. Due to GAPDH alterations, quantitative data were obtained using alternative internal controls: β-actin and total protein loading via Ponceau S staining. Lower panels display the densitometric quantification of γ-H2AX levels normalized against β-actin (top graphs) or Ponceau S (middle graphs) for both cell lines, highlighting differences between the two methods at late time points. The bottom graphs illustrate the β-actin/Ponceau S ratio, suggesting the overall stability of the structural housekeeping control. Data in the graphs are expressed as the mean ± Standard Error of the Mean (SEM) of three independent experiments. Two-way ANOVA with Tukey’s multiple comparison test was performed to analyze the effects of the two different treatment factors. * *p* < 0.05; ** *p* < 0.01; *** *p* < 0.001; **** *p* < 0.0001.

To validate whether the observed growth inhibition was directly linked to cytotoxic mechanisms, cell death was quantitatively evaluated via flow cytometry using propidium iodide (PI) staining across three time points (24, 48, and 72 h). Since PI exclusively permeates compromised cell membranes, it serves as a selective marker for late apoptotic and necrotic populations. As illustrated in Figure 5A–D, TPP25 treatment at the IC_50_ dose triggered a significant, time-dependent increase in PI fluorescence intensity (Mean Fluorescence Intensity, MFI), corresponding to a markedly higher percentage of dead cells compared to the vehicle controls. Notably, distinct sensitivity profiles emerged between the two GBM lines: U87 cells exhibited a significant susceptibility to both IC_50_ and IC_25_ doses, reaching peak cytotoxicity at 72 h (Figure 5C,D); T98 cells displayed a more restricted response, where the cytotoxic effect was predominantly prominent at the IC_50_ dose after 72 h of exposure (Figure 5A,B).

To clarify the mode of cell death and differentiate between early/late apoptosis and necrosis, an Annexin V/7-AAD dual-staining assay was performed (Figure S19). In both cell lines, TPP25 exposure led to a time- and dose-dependent shift from the viable population (Annexin V^−^/7-AAD^−^) toward early apoptotic (Annexin V^+^/7-AAD^−^) and predominantly late apoptotic/necrotic (Annexin V^+^/7-AAD^+^) stages. Consistent with the PI single-staining data, U87 cells showed higher sensitivity, with a statistically significant accumulation of Annexin V^+^/7-AAD^+^ cells observable starting from 48 h at both concentrations. Conversely, T98 cells exhibited a significant transition to late apoptosis mainly at the highest concentration at the 72 h time point.

To clarify the molecular mechanisms driving this process, caspase-3 activation was evaluated. As shown in Figure 5E, a doublet corresponding to cleaved caspase-3 was detected following exposure to the lower dose of TPP25, which suggests the initiation of an apoptotic-like cascade. Interestingly, at the higher cytotoxic dose, the caspase-3 signal appeared weaker. This non-monotonic, dose-dependent decrease may point toward an advanced stage of cell death characterized by generalized protein degradation rather than a specific downregulation of the apoptotic machinery. This hypothesis is supported by a concurrent decrease in the housekeeping control glyceraldehyde 3-phosphate dehydrogenase (GAPDH) levels at the highest concentration. This global reduction in GAPDH highlights a technical limitation under high-dose cytotoxic conditions at 72 h; therefore, the data regarding caspase-3 must be interpreted qualitatively and with caution.

To explore whether the cytotoxic response was orchestrated by severe genotoxic stress, we evaluated the expression levels of the phosphorylated form of the histone H2AX (γ-H2AX), an established biomarker for DNA double-strand breaks (DSBs). Given the observed alterations of GAPDH at higher doses, data quantification for γ-H2AX expression was strictly cross-validated and corroborated using both an alternative loading control, namely β-actin, and total protein normalization via Ponceau S staining (Figure 5F). Notably, the β-actin/Ponceau S ratio suggests that β-actin remained considerably stable across treatments, exhibiting only minor fluctuations even under high-dose conditions. Despite this overall stability, the precise magnitude and kinetics of γ-H2AX accumulation varied slightly depending on the normalization method applied, though the overarching experimental trends were consistently preserved. In T98 cells, normalization against β-actin revealed a significant upregulation of γ-H2AX at 48 h at the 11 μM dose and a sustained high-level plateau at 72 h for both treated doses. Interestingly, when normalized against total protein (Ponceau S), the 72 h accumulation of γ-H2AX at the highest dose (11 μM) appeared markedly more pronounced. In U87 cells, both normalization strategies captured a dose-dependent spike in γ-H2AX levels at 48 h and a moderate decline at the highest concentration compared to the intermediate dose. Taken together, these detailed quantitative analyses and their corresponding statistical tests suggest a strong genotoxic triggering at intermediate thresholds, while the fluctuating signal amplification at the highest cytotoxic doses further supports the hypothesis of an advanced, irreversible cell death phase characterized by differential proteolysis rates.

#### 2.2.3. Effect of TPP25 Compound on Intracellular ROS Production and Mitochondrial Membrane Potential

**Figure 6:**
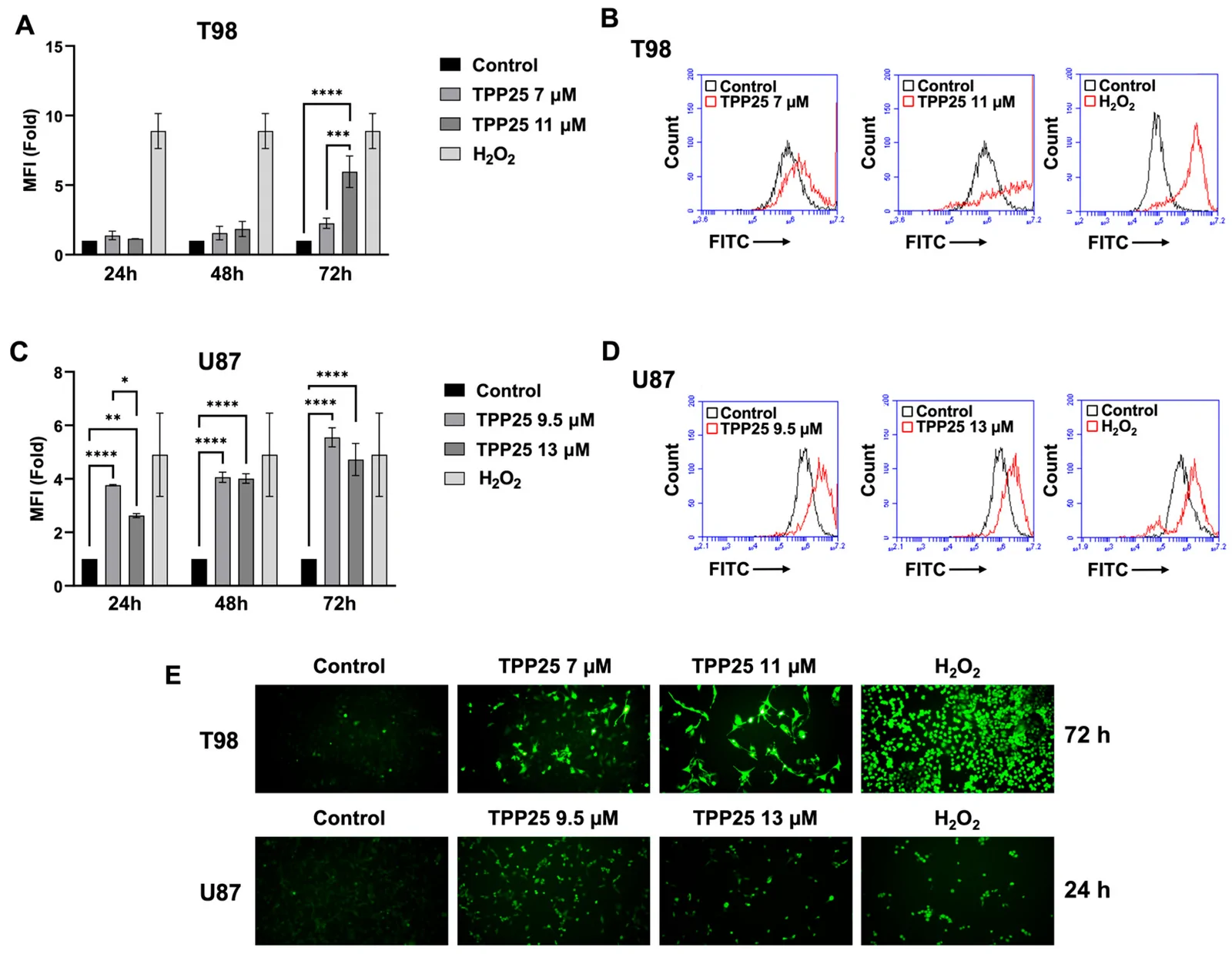
Intracellular ROS production induced by TPP25 in T98 and U87 GBM cell lines. (**A**,**C**) Quantitative flow cytometric analysis of intracellular reactive oxygen species (ROS) levels measured via 2′,7′-dichlorodihydrofluorescein diacetate (DCFDA) assay. Bar graphs represent the Mean Fluorescence Intensity (MFI) expressed as fold-change relative to control groups across three different time points for (**A**) T98 and (**C**) U87 cells. Hydrogen peroxide (H_2_O_2_) was utilized as positive control. Data are expressed as the mean ± Standard Error of the Mean (SEM) of three independent experiments. Two-way ANOVA with Tukey’s multiple comparison test was performed to analyze the individual and interactive effects of the two different treatment factors. * *p* < 0.05; ** *p* < 0.01; *** *p* < 0.001; **** *p* < 0.0001. (**B**,**D**) Representative flow cytometry overlay histograms (FITC channel) at 72 h treatment for (**B**) T98 and (**D**) U87 cells, comparing vehicle-treated control populations (black line) with TPP25-treated groups or the positive control (red line). (**E**) Representative qualitative fluorescence microscopy images of DCFDA green fluorescence showing intracellular ROS accumulation in T98 cells (after 72 h) and U87 cells (after 24 h) under control conditions, after TPP25 exposure, or upon H_2_O_2_ positive control treatment. Magnification 10×.

To elucidate whether the cytotoxic effects of TPP25 were mediated by oxidative stress, intracellular reactive oxygen species (ROS) levels were evaluated in GBM cells treated with IC_50_ and IC_25_ doses. For this purpose, cells were loaded with 2′,7′-dichlorodihydrofluorescein diacetate (DCFDA), a non-fluorescent fluorophore that undergoes deacetylation by cellular esterases and subsequent oxidation by ROS into the highly fluorescent 2′,7′-dichlorofluoroscein (DCF). Hydrogen peroxide (H_2_O_2_) was utilized as a positive control. As shown in Figure 6A–D, flow cytometric analysis revealed distinct, line-specific kinetics of ROS generation. In the U87 line, a statistically significant increase in ROS production was detected after exposure to both doses as early as 24 h. Conversely, T98 cells displayed a delayed response, with a significant increase in ROS levels observed exclusively after 72 h of treatment with the IC_50_ dose. These quantitative findings were qualitatively verified by fluorescence microscopy (Figure 6E), which demonstrated an increase in green fluorescence intensity at 24 h for U87 cells and at 72 h for T98 cells, mirroring the cytometric profiles.

**Figure 7:**
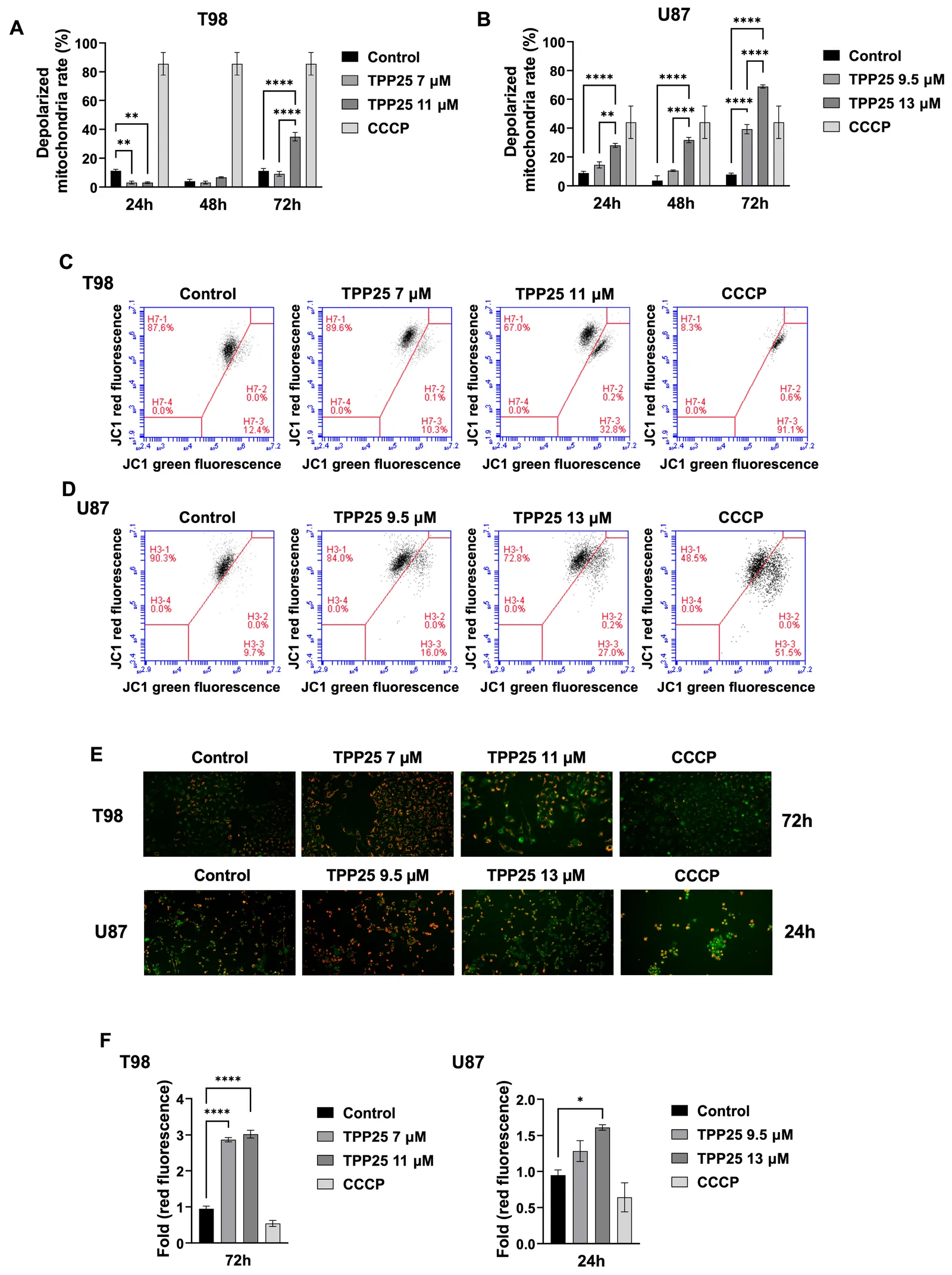
Evaluation of mitochondrial membrane potential disruption induced by TPP25 in T98 and U87 GBM cell lines. (**A**,**B**) Quantitative analysis of the percentage of cells with depolarized mitochondria evaluated via JC-1 assay across three different time points for (**A**) T98 and (**B**) U87 cells. Carbonyl cyanide m-chlorophenyl hydrazone (CCCP) was utilized as a positive control for mitochondrial depolarization. Data are expressed as the mean ± Standard Error of the Mean (SEM) of three independent experiments. Two-way ANOVA with Tukey’s multiple comparison test was performed to analyze the individual and interactive effects of the two different treatment factors. ** *p* < 0.01; **** *p* < 0.0001. (**C**,**D**) Representative flow cytometric dot plots showing the shift from JC-1 aggregates (high potential, upper left quadrant) to monomers (depolarized potential, lower right quadrant) at the indicated treatment time points for (**C**) T98 (72 h) and (**D**) U87 (24 h) cells. (**E**) Representative qualitative fluorescence microscopy images of JC-1 staining in T98 (72 h) and U87 (24 h) cells. Red/orange fluorescence indicates healthy mitochondria with high potential (JC-1 aggregates), while green fluorescence indicates depolarized mitochondria (JC-1 monomers). (**F**) Bar graphs quantifying the ratio or fold-change in red fluorescence in T98 and U87 cells, highlighting the transient mitochondrial hyperpolarization phase prior to collapse. Magnification 10×. Data are expressed as the mean ± Standard Error of the Mean (SEM) of three independent experiments. One-way ANOVA with Dunnett’s multiple comparison test was performed to compare each treatment group specifically against control. * *p* < 0.05; **** *p* < 0.0001.

To investigate whether this oxidative stress was coupled with mitochondrial dysfunction, changes in the mitochondrial membrane potential (ΔΨ_m_) were assessed using the JC-1 cationic dye across 24, 48, and 72 h. Carbonyl cyanide m-chlorophenyl hydrazone (CCCP) served as the positive control for depolarization. In healthy mitochondria with a high membrane potential, JC-1 selectively concentrates to form aggregates that emit a red fluorescence. Conversely, in depolarized or damaged mitochondria, JC-1 remains in its monomeric form in the cytoplasm, emitting a green fluorescence. Flow cytometric analysis (Figure 7A–D), which monitors the percentage of cells with depolarized mitochondria, demonstrated that TPP25 initiated mitochondrial depolarization at the sublethal IC_25_ dose in U87 cells within 24 h, progressing to a marked drop in ΔΨ_m_ at the IC_50_ dose. In contrast, T98 cells displayed an early response at 24 h, characterized by a significant decrease in baseline depolarization (reflecting a transient hyperpolarization phase), and successfully resisted mitochondrial breakdown until 72 h of exposure. Interestingly, these data match the kinetics of PI incorporation and late-stage ROS production previously observed. These results were further substantiated by fluorescence microscopy (Figure 7E). Notably, both cytometric analysis (Figure 7C,D,F) and microscopic imaging (Figure 7E) revealed a hyperpolarization phase. This apparent divergence can be attributed to cell subpopulation heterogeneity within the non-synchronous treated cultures; while a substantial fraction of cells has committed to cell death and exhibits a compromised mitochondrial potential, the remaining viable or transiently resistant subpopulation still undergoes this compensatory, stress-induced mitochondrial hyperpolarization as an adaptive survival mechanism.

## 3. Discussion

The high heterogeneity of GBM, combined with multiple drug-related resistance mechanisms, contributes to the limited success of current therapeutic strategies and makes the development of novel chemical scaffolds an urgent challenge [1]. In this work, the newly synthesized pyrazolone-based hydrazone derivative, TPP25, emerged as the most promising compound of the series. Chemically, its performance is strongly driven by the trifluoromethyl group that gives the molecule a particular electronic distribution [17,18,19]. Furthermore, the high lipophilicity of the molecule facilitates its passive penetration across lipid bilayers and biological membranes. These unique electronic and lipophilic properties optimize plasma membrane permeation and may favor a passive partition toward the mitochondrial compartment, offering a tentative rationale for the mitochondrial distress observed in our assays. However, whether this structural profile translates into selective mitochondrial targeting or preferential accumulation in malignant cells remains a preliminary hypothesis. Further compartmentalization analyses and comparative biophysical studies are required to fully elucidate the intracellular distribution of TPP25, representing a primary objective of our future research.

In our preliminary screenings, TPP25 displayed a promising in vitro selectivity window against the non-tumoral NHF A12 cell line. The absence of cytotoxicity against healthy fibroblasts up to the maximum tested concentration (20 μM) allowed us to define a minimum Selectivity Index (SI) of >1.8 for T98 cells and >1.5 for U87 cells. In the context of neuro-oncology, achieving high selectivity against malignant phenotypes is critical to avoid treatment-induced neural damage. These data strongly support the therapeutic window of TPP25, though further investigations on normal human astrocytes will be essential to fully validate its safety profile in the central nervous system microenvironment. Furthermore, our assays exposed a contrast in how different genetic backgrounds process TPP25-induced stress, revealing distinct phenotypic behaviors that do not preclude apoptotic commitment in either model. In p53 wild-type U87 cells, the compound induced a time-dependent cell death, without triggering a conventional cell cycle arrest. Conversely, in the p53-mutant T98 line, cells lacked a functional G1 checkpoint and continuously replicated under chemical distress until reaching a lethal threshold that triggered a sudden entry into the sub-G0 phase. These observations suggest that TPP25 can activate downstream death pathways regardless of the baseline p53 status. This cytotoxic response was orchestrated by severe genotoxic stress, as supported by the robust dose-dependent upregulation of the phosphorylated form of the histone γ-H2AX at 48 h in both cell lines. However, a detailed cross-validation using β-actin and Ponceau S total protein normalization unmasked distinct, line-specific kinetics of DNA damage accumulation at 72 h. In T98 cells, genotoxic stress was cumulative and sustained; β-actin normalization showed a high-level plateau at 72 h, while Ponceau S normalization revealed an even more pronounced 50-fold increase at 11 μM. Conversely, U87 cells showed a sharp 72 h collapse of γ-H2AX. This signal disappearance points toward advanced cell death.

The kinetics of intracellular ROS generation mirrored the line-specific mortality rates, showing an early oxidative increase in U87 cells and a delayed accumulation in T98 cells. Our JC-1 assays provided critical insight into this bioenergetic collapse, revealing a transient mitochondrial hyperpolarization phase, particularly visible at the sublethal dose, prior to the final loss of ΔΨ_m_. This profile represents a classic hallmark of cell subpopulation heterogeneity within a non-synchronous culture subjected to severe chemical stress. The treated population is biologically split: while a substantial subpopulation has entered the definitive execution phase of cell death (accounting for the increased depolarization rate), the remaining viable or transiently resistant subpopulation initiates an adaptive hyperpolarization of intact mitochondria to maintain ATP production. This temporary increase in membrane potential represents an early, compensatory metabolic adaptation where mitochondria hyperactivate the electron transport chain to resist the toxic insult; however, this state accelerates electron leaks and ROS generation, establishing an oxidative feedback loop that ultimately leads to complete depolarization. The subsequent release of pro-apoptotic factors into the cytosol likely contributes to the transient cleavage of Caspase-3, suggesting the engagement of an active apoptotic cascade before total cellular collapse. Intriguingly, while this active caspase cascade led to a highly pronounced sub-G0 population in T98 cells, the apoptotic-like U87 cells exhibited a robust loss of membrane integrity (high live-cell PI uptake) without a corresponding hypodiploid peak. This apparent divergence can be reconciled by well-documented cell-line-specific differences in DNA fragmentation mechanics within glioblastoma models. In standard apoptosis, active Caspase-3 cleaves Inhibitor of Caspase-Activated DNase (ICAD) to release Caspase-Activated DNase (CAD/DFF40), which executes the internucleosomal cleavage required to generate a sub-G0 population [20]. However, as demonstrated by Sanchez-Osuna et al. [21], an intrinsic deficiency or low expression of DFF40/CAD endonuclease is a widespread trait among human glioblastoma cell lines, including U87, which selectively impairs oligonucleosomal DNA hydrolysis during caspase-dependent cell death. Consequently, while apoptotic-like hallmarks (Caspase-3 cleavage and Annexin V positivity) and membrane permeabilization (PI uptake) proceed efficiently, the DNA cleavage is likely arrested at large-molecular-weight fragments, thereby preventing the accumulation of a distinct hypodiploid fraction in U87 cells despite their validated apparent apoptotic involvement [22].

In conclusion, by leveraging its optimized, lipophilic structure, TPP25 effectively exploits mitochondrial and DNA vulnerabilities in vitro, exhibiting strong cytotoxicity against both p53-wild-type and p53-mutant GBM models. Despite these encouraging results, evaluating selectivity against a single non-tumoral cell type represents only an initial screening step and is insufficient to prove overall biocompatibility or systemic safety. Therefore, future prospects will focus on conducting functional validation through specific caspase inhibitors, alongside comprehensive toxicity profiles across a broader panel of non-tumoral models, including normal astrocytes and endothelial cells. Furthermore, since the therapeutic efficacy of new anti-GBM compounds ultimately depends on their ability to cross the blood–brain barrier, dedicated penetration assays will be required to validate their central nervous system availability. Subsequent formulation strategies will also be pursued to encapsulate TPP25 within advanced nanodelivery systems, thereby aiming to maximize its concentration at the tumor site while minimizing systemic side effects.

## 4. Materials and Methods

### 4.1. General Experimental Procedures

All reagents and solvents were acquired from Sigma-Aldrich Chemical Company (Darmstadt, Germany). Compounds were characterized by ^1^H NMR, ^13^C NMR, MS, and elemental analyses. ^1^H (500 MHz) and ^13^C (126 MHz) spectra of the synthesized compounds were obtained using a Varian Mercury 500 (Varian, Inc., Palo Alto, CA, USA); chemical shift values were expressed in *δ* values (ppm) and coupling constants (*J*) in Hertz. Melting points were taken in glass capillary tubes on a Büchi SMP-20 apparatus (Büchi Laboratory Equipment, Uster, Switzerland). Mass spectra were recorded using an Agilent HP 1100 series instrument (Agilent Technologies, Santa Clara, CA, USA). All measurements were performed in positive ion mode using atmospheric pressure electrospray ionization (API-ESI). Elemental analyses (C, H, and N) were performed using a ThermoFisher Scientific FLASH 2000 CHNS analyzer (Thermo Fisher Scientific, Waltham, MA, USA). All the synthesized compounds were >95% pure by NMR analysis.

### 4.2. General Synthesis

The 4-acylpyrazolone ligand 1-(5-hydroxy-3-methyl-1-phenyl-1*H*-pyrazol-4-yl)-2-phenylethan-1-one (HQ^Ph,Bn^) was synthesized following the method reported by Marchetti et al. [10]. The TPPs were synthesized as follows: To a solution of HQ^Ph,Bn^ (1.0 equiv.) in methanol (10 mL), the appropriate hydrazine (1.0 equiv.) was added along with 5 drops of glacial acetic acid. The mixture was heated to 80 °C for different times (2–4 h). Reaction progress was monitored by thin-layer chromatography. A precipitate slowly formed from the hot solution, and, after completion, the reaction mixture was stored at 4 °C overnight. The obtained precipitate was filtered and dissolved in ethanol (10 mL). A solid was obtained by slow evaporation of the solvent, then washed with methanol and diethyl ether, and dried under vacuum.

**Scheme 2:**
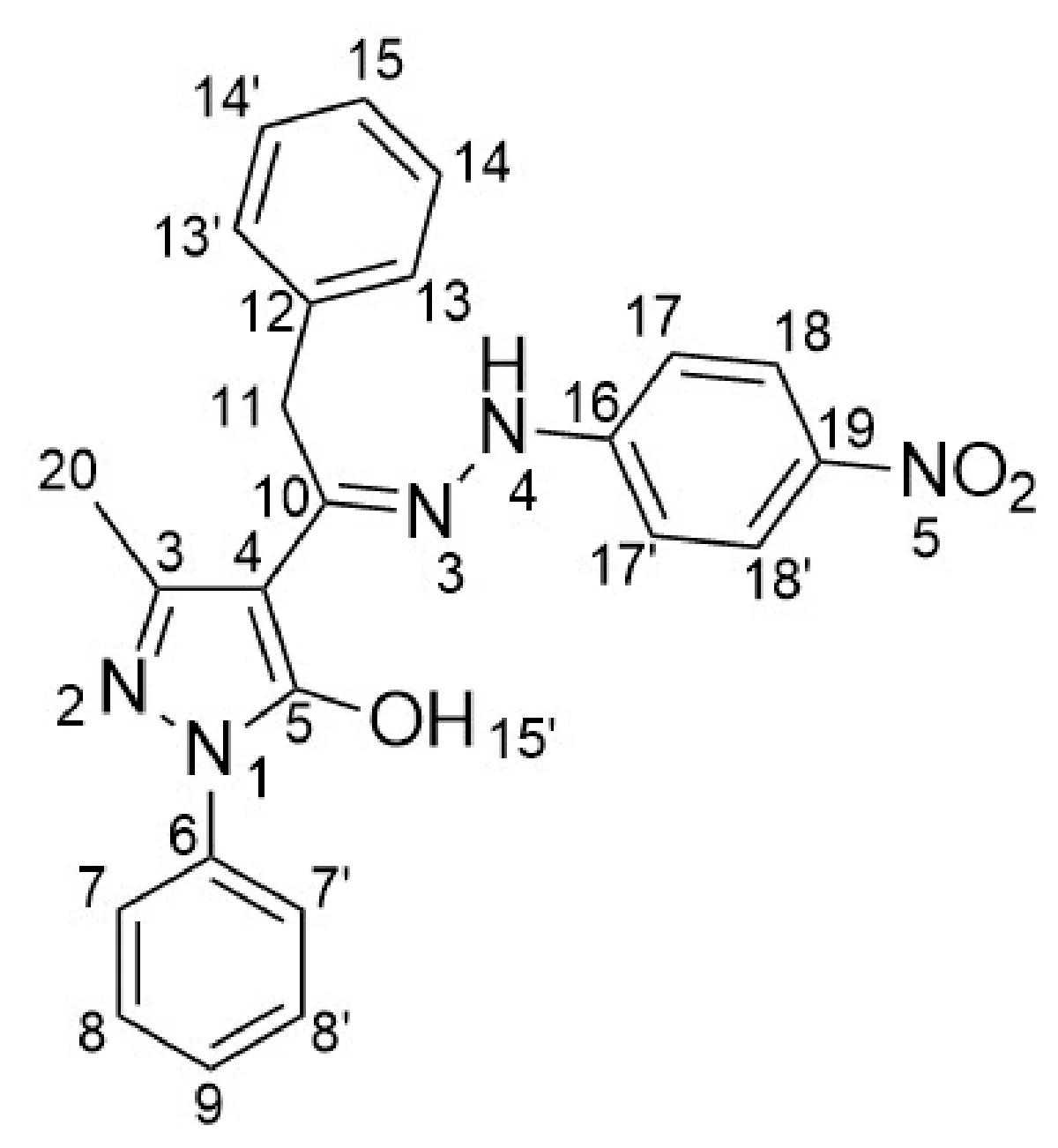
Compound TPP08.

Compound TPP08. (Scheme 2) 3-methyl-4-((E)-1-(2-(4-nitrophenyl)hydrazineylidene)-2-phenylethyl)-1-phenyl-1*H*-pyrazol-5-ol. TPP08 was synthesized from HQ^Ph,Bn^ (254 mg, 0.87 mmol) and (4-nitrophenyl)hydrazine hydrochloride following the general procedure previously described (reaction time 4 h) and as reported by Marchetti et al. [23]. Brown solid (269 mg, yield: 72%), mp 228–229 °C. ^1^H NMR (500 MHz, CDCl_3_) *δ* 12.44 (brs, 1H, O−*H*), 8.08 (d, *J* = 9.1 Hz, 2H, *H*18,18′), 7.99 (d, *J* = 7.4 Hz, 2H, *H*7,7′), 7.43 (t, *J* = 8.6, 7.4 Hz, 2H, *H*8,8′), 7.32–7.27 (m, 3H, *H*14,14′, *H*9), 7.21 (t, *J* = 7.4, 1.2 Hz, 1H, *H*15), 7.17 (d, *J* = 6.9 Hz, 2H, *H*13,13′), 6.68 (d, *J* = 9.1 Hz, 2H, *H*17,17′), 6.32 (s, 1H, N4-*H*), 4.13 (s, 2H, *H*11), 2.41 (s, 3H, *H*20); ^13^C NMR (126 MHz, CDCl_3_) *δ* 166.92, 165.38, 151.50, 147.12, 141.97, 138.72, 134.34, 129.50, 129.05, 128.08, 127.79, 126.04, 125.23, 119.66, 112.05, 100.99, 33.84, 16.86; ESI-MS (+) CH_3_CN *m*/*z* = 428 [TPP08 + H]^+^; 450 [TPP08 + H + Na]^+^; 491 [TPP08 + H + CH_3_CN + Na]^+^; 878 [2TPP08 + H + Na]^+^; Anal. calcd for C_24_H_21_N_5_O_3_: C, 67.44; H, 4.95; N, 16.38%. Found: C, 67.23; H, 4.83; N, 16.11%.

**Scheme 3:**
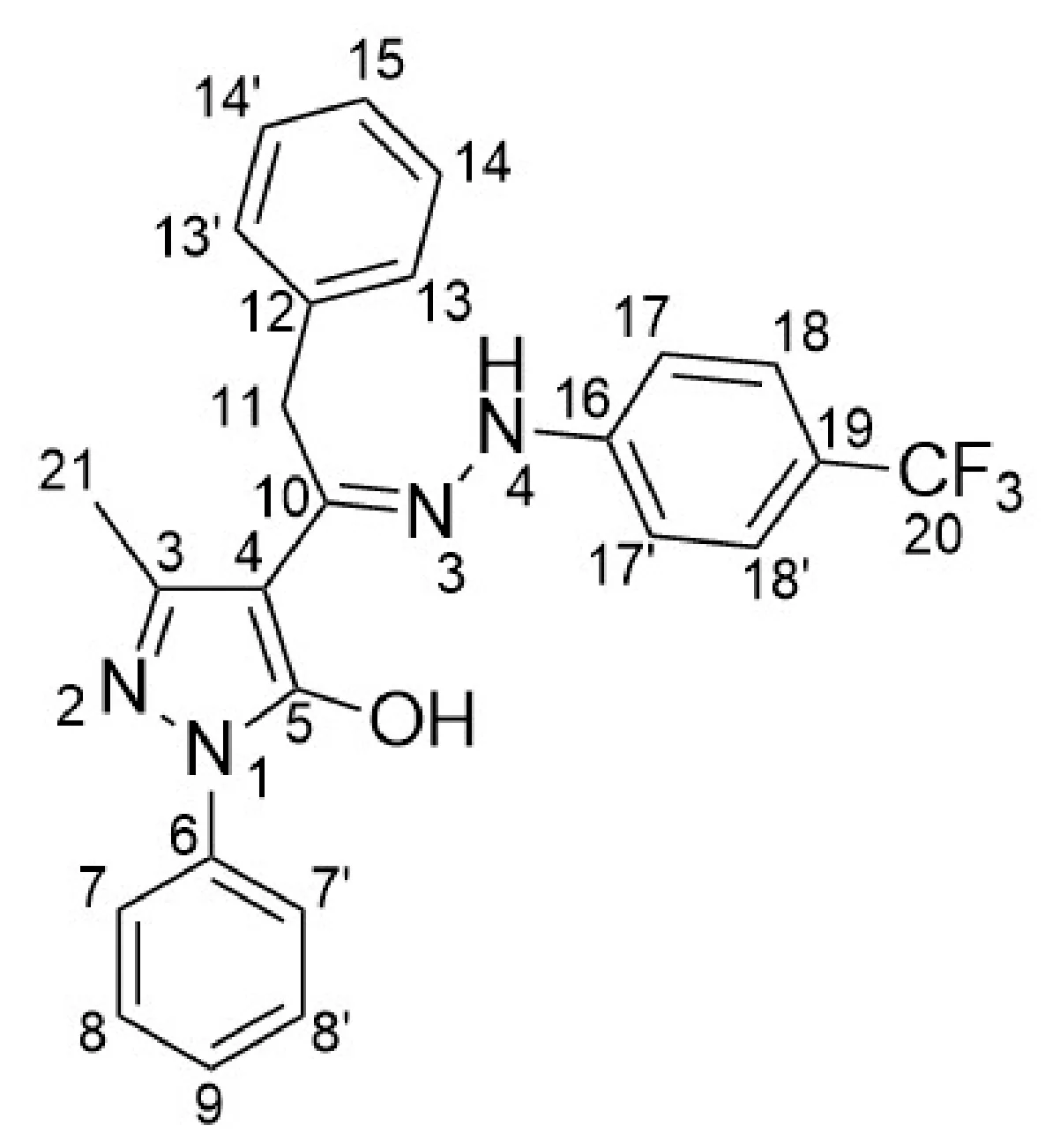
Compound TPP25.

Compound TPP25. (Scheme 3) 3-methyl-1-phenyl-4-((E)-2-phenyl-1-(2-(4-(trifluoromethyl)phenyl)hydrazineylidene)ethyl)-1*H*-pyrazol-5-ol. TPP25 was synthesized from HQ^Ph,Bn^ (506 mg, 1.73 mmol) and 4-(trifluoromethyl)phenylhydrazine, following the general procedure previously described (reaction time 2 h) and as reported by Marchetti et al. [10]. Yellow solid (531 mg, yield: 68%), mp 191–192 °C. ^19^F NMR was not recorded as TPP25 was previously characterized in Marchetti et al. [10]; identity/purity confirmed by ^1^H/^13^C NMR, MS, elemental analysis. ^1^H NMR (500 MHz, CDCl_3_) *δ* 12.47 (brs, 1H, O−*H*), 8.02 (d, *J* = 7.4 Hz, 2H, *H*7,7′), 7.47 (d, *J* = 8.6 Hz, 2H, *H*18,18′), 7.44 (t, *J* = 8.0 Hz, 2H, *H*8,8′), 7.37–7.28 (m, 3H, *H*14,14′, *H*15), 7.23–7.18 (m, 3H, *H*13,13′, *H*9), 6.77 (d, *J* = 8.6 Hz, 2H, *H*17,17′), 6.00 (s, 1H, N4-*H*), 4.19 (s, 2H, *H*11), 2.42 (s, 3H, *H*21); ^13^C NMR (126 MHz, CDCl_3_) *δ* 167.21, 165.64, 148.84, 146.96, 138.75, 134.59, 129.28, 128.81, 127.94, 127.47, 126.90q (^3^*J*_C−F_ = 3.9 Hz), 125.30q (^1^*J*_C−F_ = 270.6 Hz), 124.77, 123.58q (^2^*J*_C−F_ = 32.9 Hz) 119.34, 112.62, 100.45, 33.58, 16.74; ESI-MS (+) CH_3_CN *m*/*z* = 451 [TPP25 + H]^+^; 473 [TPP25 + H + Na]^+^; 489 [TPP25 + H + K]^+^; Anal. calcd for C_25_H_21_F_3_N_4_O; C, 66.66; H, 4.70; N, 12.44%. Found: C, 66.31; H, 4.58; N, 12.74%.

**Scheme 4:**
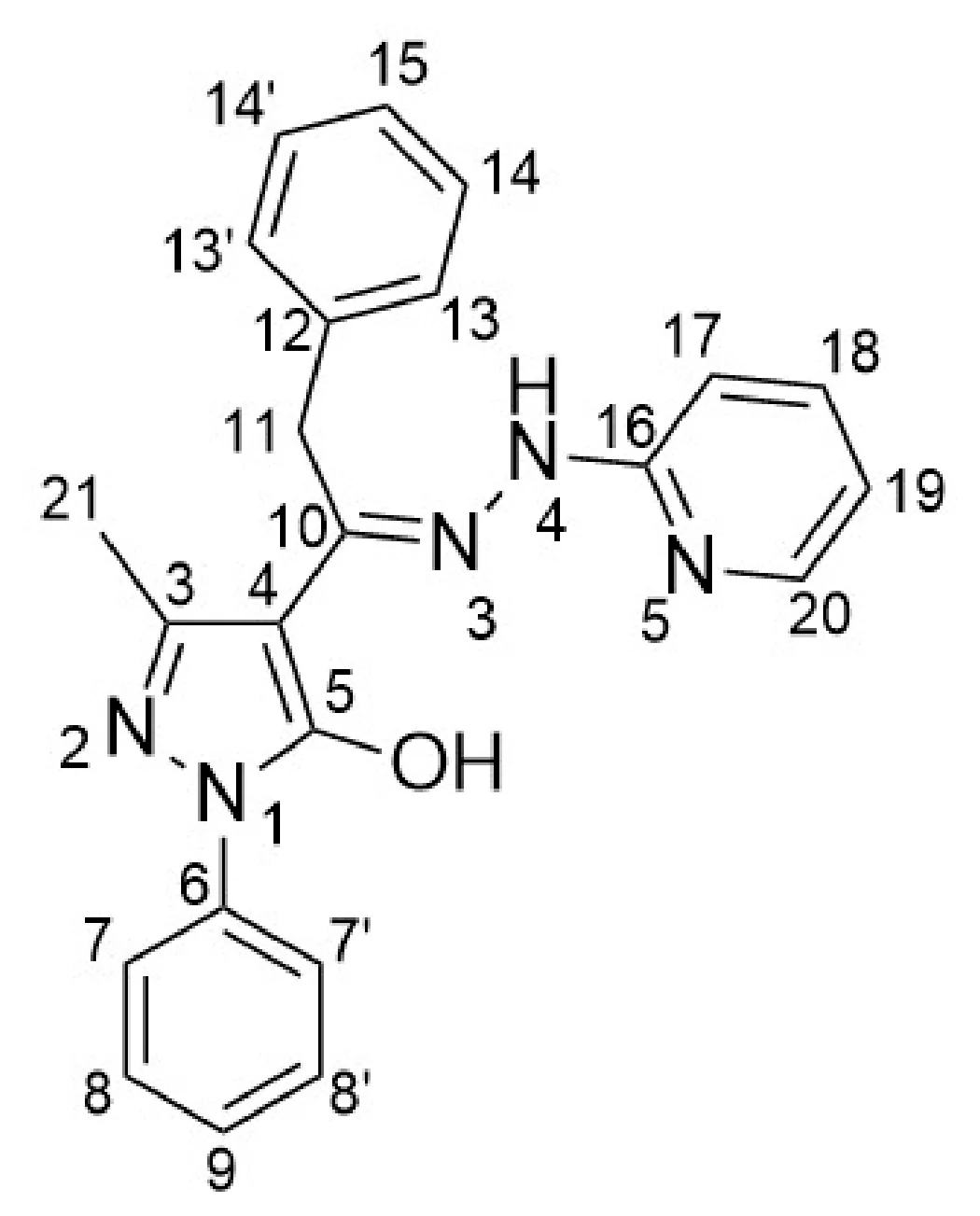
Compound TPP26.

Compound TPP26. (Scheme 4) 3-methyl-1-phenyl-4-((Z)-2-phenyl-1-(2-(pyridin-2-yl)hydrazineylidene)ethyl)-1*H*-pyrazol-5-ol. TPP26 was synthesized from HQ^Ph,Bn^ (392 mg, 1.34 mmol) and 2-hydrazinopyridine, following the general procedure previously described (reaction time 2 h) and as reported by Marchetti et al. [10]. Yellow solid (333 mg, yield: 65%), mp 240–241 °C. ^1^H NMR (500 MHz, CDCl_3_) *δ* 12.55 (brs, 1H, O−*H*), 8.10 (d, *J* = 3.3 Hz, 1H, *H*20), 8.04 (d, *J* = 7.4 Hz, 2H, *H*7,7′), 7.49 (t, *J* = 6.9 Hz, 1H, *H*18), 7.43 (t, *J* = 7.0 Hz, 2H, *H*8,8′), 7.29 (t, J = 7.6 Hz, 2H, *H*13,13′), 7.27–7.20 (m, 3H, *H*14,14′, *H*15), 7.19 (t, *J* = 7.4 Hz, 1H, *H*9), 6.82 (t, *J* = 7.3 Hz, 1H, *H*19), 6.56 (d, J = 8.3 Hz, 1H, *H*17), 4.22 (s, 2H, *H*11), 2.42 (s, 3H, H21), (the hydrazone N–H proton was not observed due to severe broadening from rapid proton exchange); ^13^C NMR (126 MHz, CDCl_3_) *δ* 167.12, 165.63, 157.83, 148.16, 146.98, 138.85, 138.44, 134.59, 129.13, 128.74, 128.07, 127.27, 124.57, 119.26, 117.19, 106.73, 100.15, 33.60, 16.74; ESI-MS (+) CH_3_CN *m*/*z* = 384 [TPP26 + H]^+^; 407 [TPP26 + H + Na]^+^; 423 [TPP26 + H + K]^+^; Anal. calcd for C_23_H_21_N_5_O; C, 72.04; H, 5.52; N, 18.26%. Found: C, 71.96; H, 5.41; N, 18.33%.

**Scheme 5:**
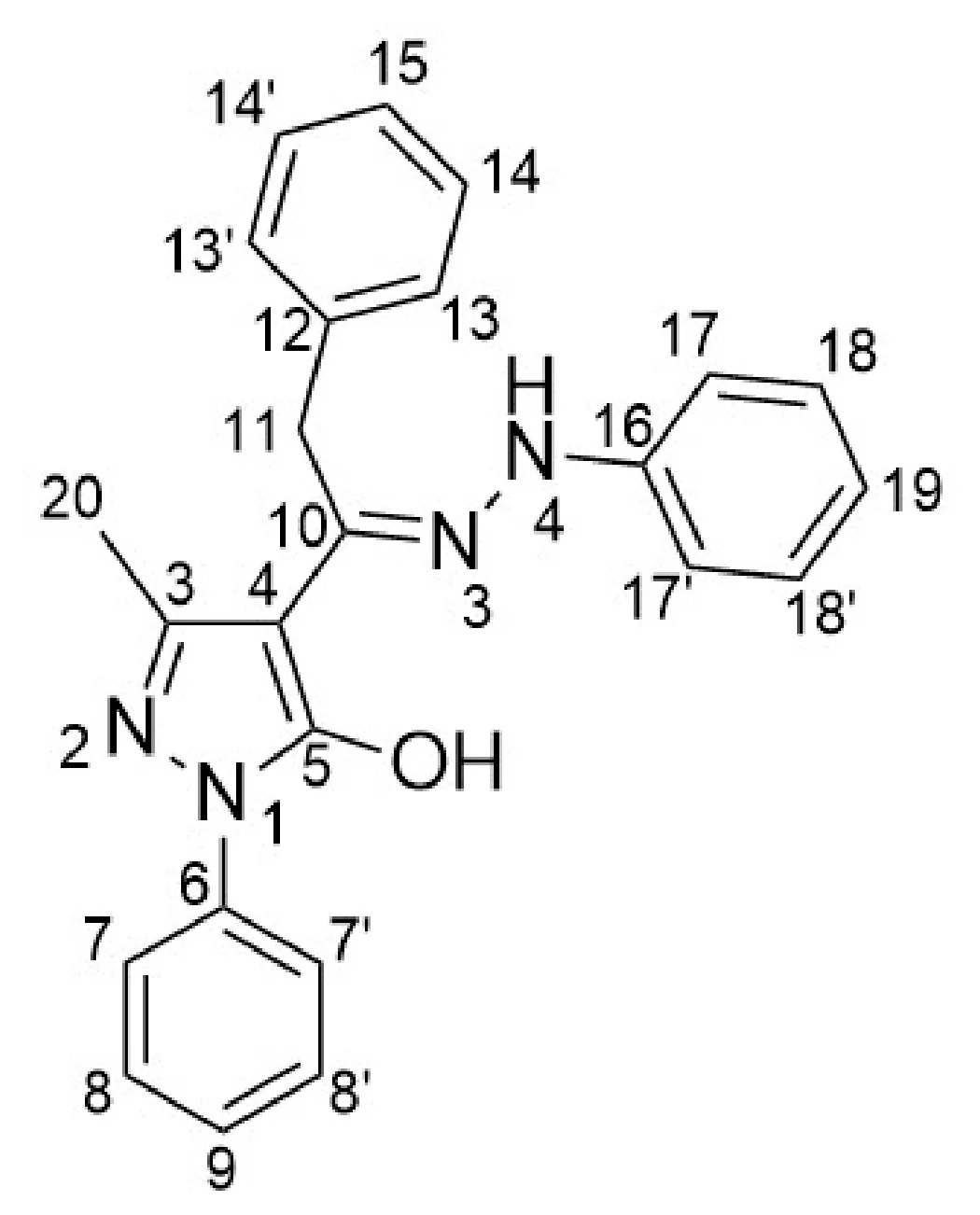
Compound TPP55.

Compound TPP55. (Scheme 5) 3-methyl-1-phenyl-4-((E)-2-phenyl-1-(2-phenylhydrazineylidene)ethyl)-1*H*-pyrazol-5-ol. TPP55 was synthesized from HQ^Ph,Bn^ (394 mg, 1.35 mmol) and phenylhydrazine, following the general procedure previously described (reaction time 4 h). White solid (506 mg, yield: 98%), mp 218–219 °C. ^1^H NMR (500 MHz, CDCl_3_) *δ* 12.46 (brs, 1H, O−*H*), 8.02 (d, *J* = 7.5 Hz, 2H, *H*7,7′), 7.41 (t, *J* = 7.1 Hz, 2H, *H*18,18′), 7.33 (t, *J* = 7.3 Hz, 2H, *H*8,8′), 7.29–7.21 (m, 5H, *H*13,13′, *H*14,14′, *H*19), 7.17 (t, *J* = 7.3, 1.2 Hz, 1H, *H*15), 6.95 (t, *J* = 7.4 Hz, 1H, *H*9), 6.76 (d, *J* = 7.7 Hz, 2H, *H*17,17′), 5.66 (s, 1H, N4-*H*), 4.23 (s, 2H, *H*11), 2.38 (s, 3H, *H*20); ^13^C NMR (126 MHz, CDCl_3_) *δ* 167.58, 166.00, 147.15, 146.13, 139.09, 135.17, 129.69, 129.35, 128.91, 128.13, 127.45, 124.68, 122.28, 119.40, 113.52, 99.98, 33.66, 16.91; ESI-MS (+) CH_3_CN *m*/*z* = 383 [TPP55 + H]^+^; 405 [TPP55 + H + Na]^+^; 446 [TPP55 + H + CH_3_CN + Na]^+^; Anal. calcd for C_24_H_22_N_4_O: C, 75.37; H, 5.80; N, 14.65%. Found: C, 75.28; H, 5.77; N, 14.68%.

**Scheme 6:**
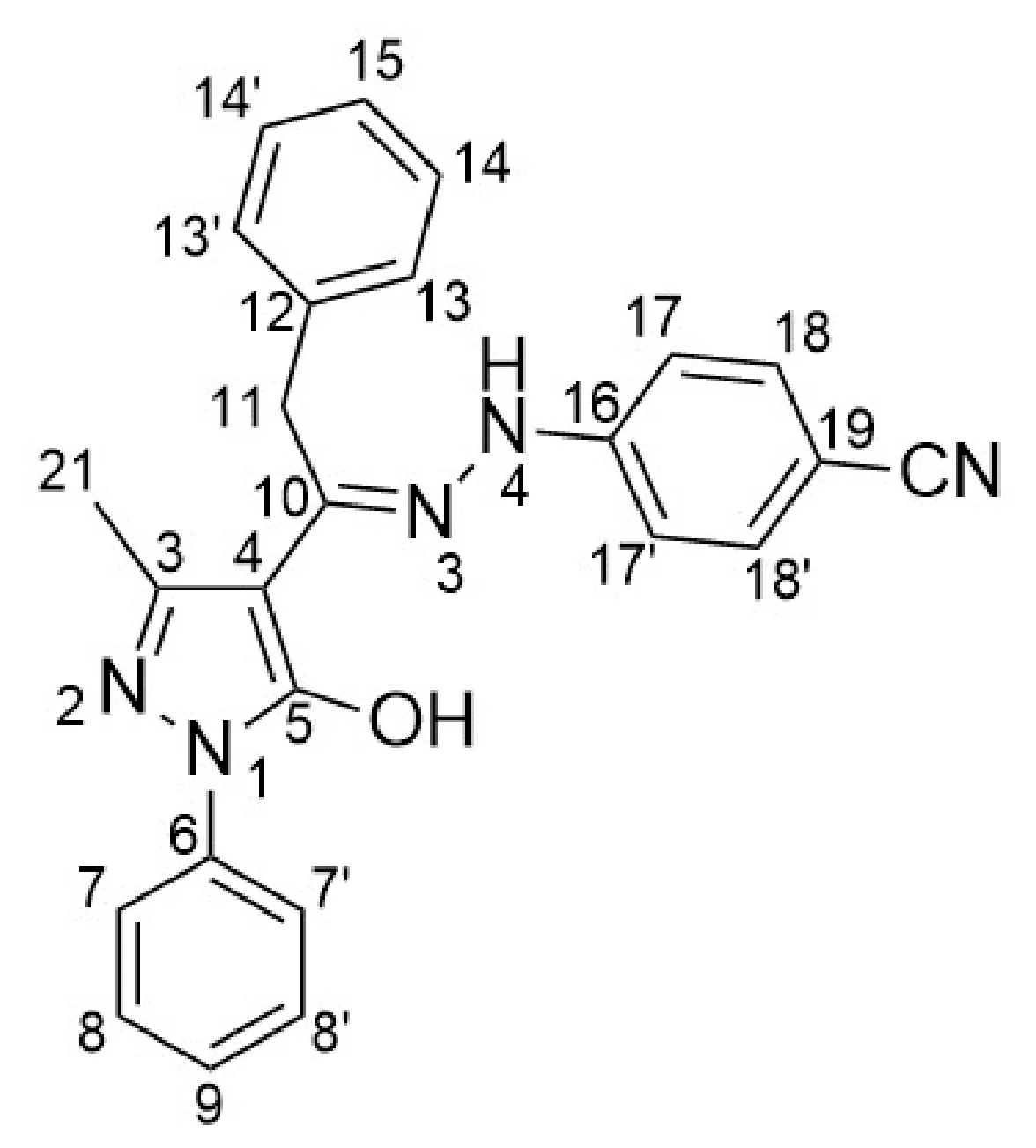
Compound TPP60.

Compound TPP60. (Scheme 6) 3-methyl-1-phenyl-4-((E)-2-phenyl-1-(2-(4-(cyano)phenyl)hydrazineylidene)ethyl)-1*H*-pyrazol-5-ol. TPP60 was synthesized from HQ^Ph,Bn^ (297 mg, 1.02 mmol) and 4-cyanophenylhydrazine hydrochloride, following the general procedure previously described (reaction time 4 h). Yellow solid (236 mg, yield: 57%), mp 238–239 °C. ^1^H NMR (500 MHz, CDCl_3_) *δ* 12.43 (brs, 1H, O−*H*), 7.99 (d, *J* = 7.5 Hz, 2H, *H*7,7′), 7.47 (d, *J* = 8.8 Hz, 2H, *H*18,18′), 7.42 (t, *J* = 7.8 Hz, 2H, *H*8,8′), 7.34–7.26 (m, 3H, *H*14,14′, *H*15), 7.23–7.15 (m, 3H, *H*13,13′, *H*9), 6.71 (d, *J* = 8.9 Hz, 2H, *H*17,17′), 6.09 (s, 1H, N4-*H*), 4.14 (s, 2H, *H*11), 2.41 (s, 3H, *H*21); ^13^C NMR (126 MHz, CDCl_3_) *δ* 167.06, 165.57, 149.78, 147.09, 138.80, 134.50, 133.95, 129.47, 129.01, 128.10, 127.73, 125.10, 119.57, 119.16, 112.96, 104.59, 100.89, 33.80, 16.88; ESI-MS (+) CH_3_CN *m*/*z* = 408 [TPP60 + H]^+^; 471 [TPP60 + H + CH_3_CN + Na]^+^; Anal. calcd for C_25_H_21_N_5_O: C, 73.69; H, 5.19; N, 17.19%. Found: C, 73.62; H, 5.15; N, 17.11%.

**Scheme 7:**
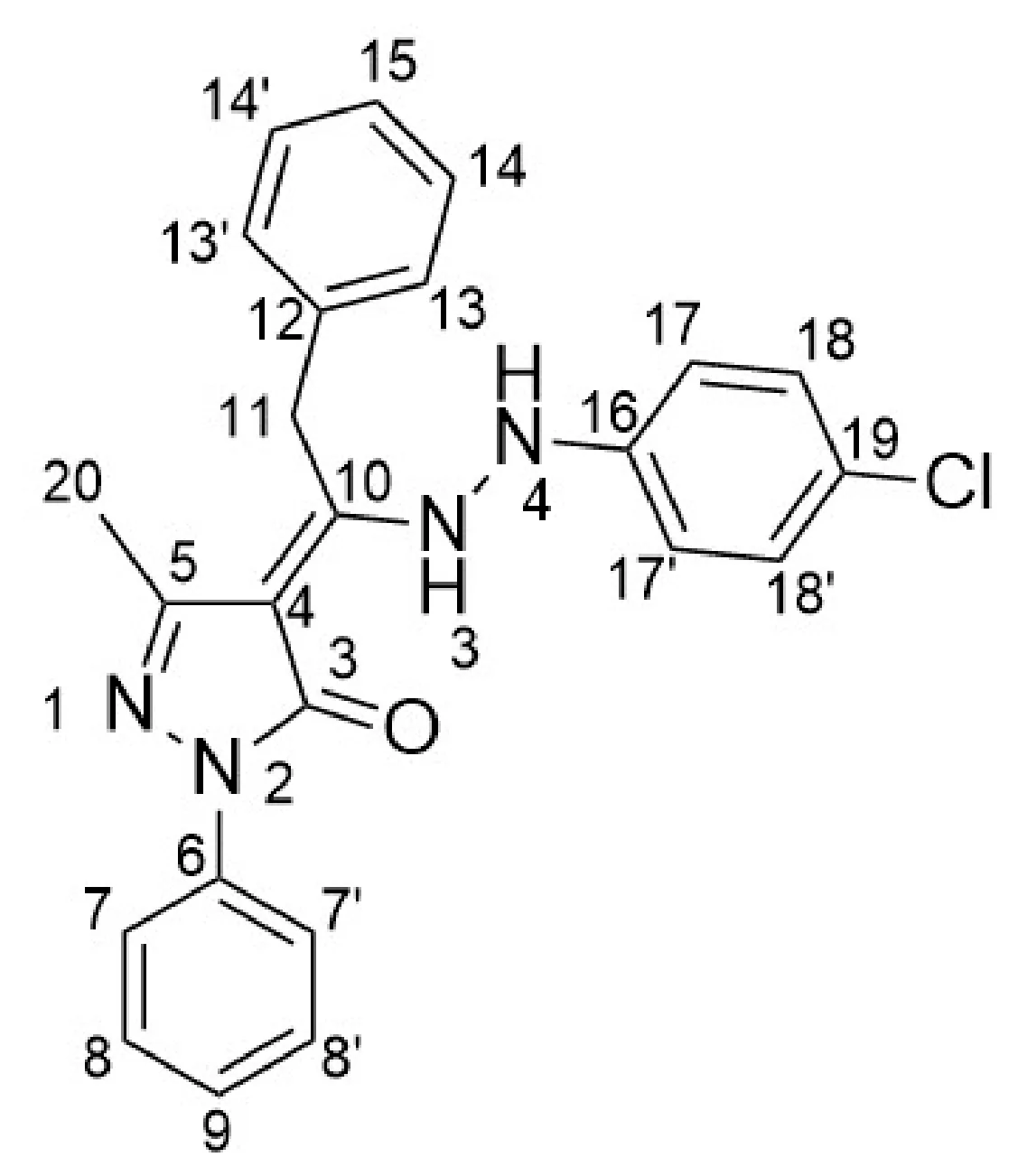
Compound TPP62.

Compound TPP62. (Scheme 7) (Z)-4-(1-(2-(4-chlorophenyl)hydrazineyl)-2-phenylethylidene)-5-methyl-2-phenyl-2,4-dihydro-3*H*-pyrazol-3-one. TPP62 was synthesized from HQ^Ph,Bn^ (297 mg, 1.02 mmol) and 4-chlorophenylhydrazine hydrochloride, following the general procedure previously described (reaction time 4 h). Yellow solid (187 mg, yield: 44%), mp 157–158 °C. ^1^H NMR (500 MHz, CDCl_3_) *δ* 7.38 (d, *J* = 8.8 Hz, 2H, *H*7,7′), 7.34–7.26 (m, 4H, *H*18,18′, *H*8,8′), 7.23–7.15 (m, 3H, *H*14,14′, *H*15), 7.14–7.07 (m, 3H, *H*13,13′, *H*9), 6.74 (d, *J* = 7.4 Hz, 2H, *H*17,17′), 4.75 (s, 2H, N3-*H*, N4-*H*), 4.01 (s, 2H, *H*11), 1.83 (s, 3H, *H*20); ^13^C NMR (126 MHz, CDCl_3_) *δ* 164.75, 151.92, 142.84, 139.01, 138.91, 137.65, 134.02, 129.44, 129.26, 128.56, 128.55, 126.65, 126.43, 126.26, 125.08, 115.50, 33.58, 11.95; ESI-MS (+) CH_3_CN *m*/*z* = 417 [TPP62 + H]^+^; 419 [TPP62 + H + 2]^+^ for ^37^Cl isotopologue (≈3:1, 417:419); 480 [TPP62 + H + CH_3_CN + Na]^+^; Anal. calcd for C_24_H_21_ClN_4_O: C, 69.14; H, 5.08; N, 13.44%. Found: C, 69.08; H, 5.02; N, 13.41%.

**Scheme 8:**
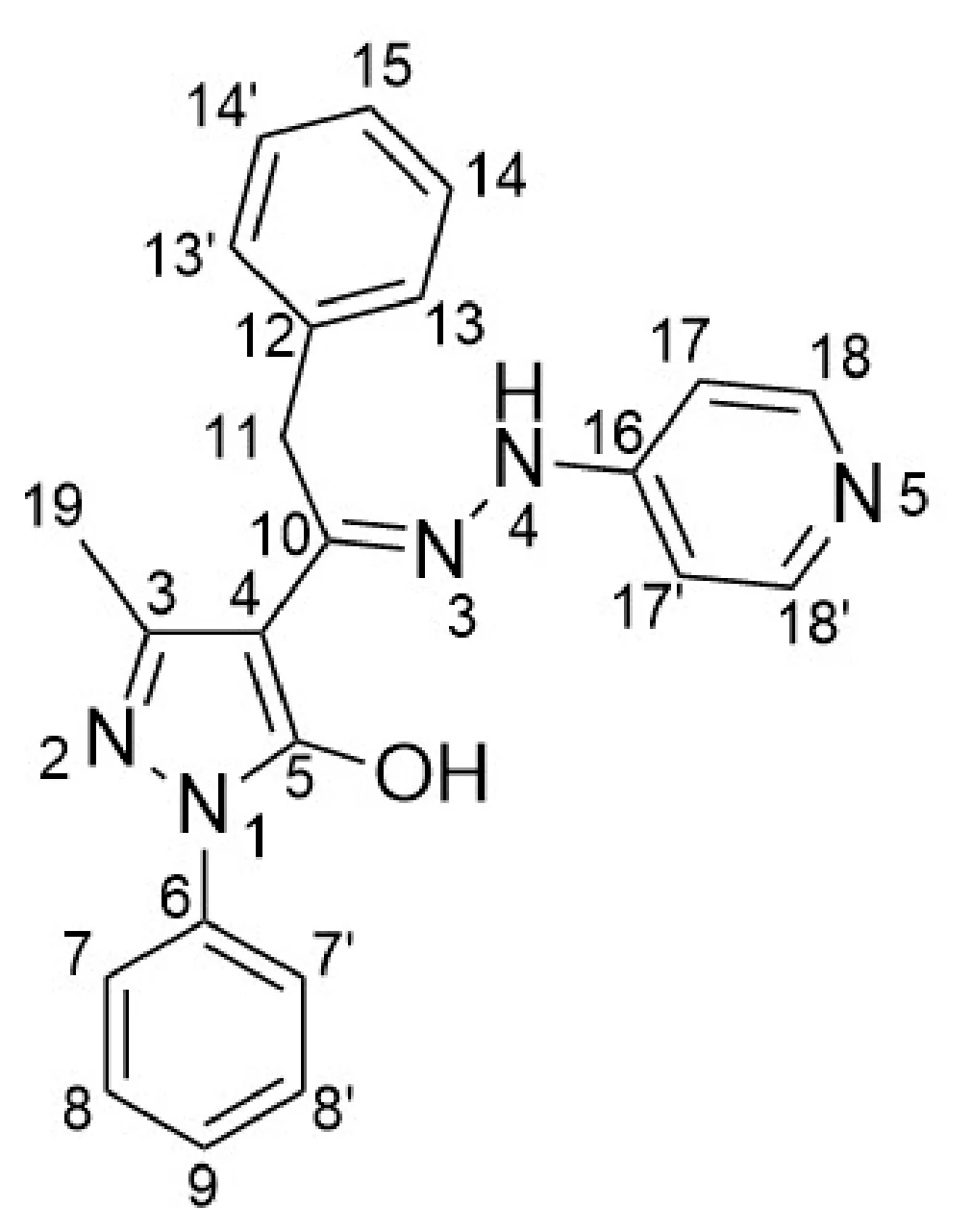
Compound TPP95.

Compound TPP95. (Scheme 8) 3-methyl-1-phenyl-4-((E)-2-phenyl-1-(2-(pyridin-4-yl)hydrazineylidene)ethyl)-1*H*-pyrazol-5-ol. TPP95 was synthesized from HQ^Ph,Bn^ (149 mg, 0.51 mmol) and 4-hydrazinopyridine hydrochloride, following the general procedure previously described (reaction time 4 h). Yellow solid (58 mg, yield: 30%), mp 238–239 °C. ^1^H NMR (500 MHz, CDCl_3_) *δ* 13.04 (brs, 1H, O−*H*), 7.87 (d, *J* = 8.1 Hz, 2H, *H*18,18′), 7.43 (t, *J* = 7.8 Hz, 2H, *H*8,8′), 7.34–7.11 (m, 10H, *H*7,7′, *H*14,14′, *H*15, *H*13,13′, *H*9, *H*17,17′), 4.28 (s, 2H, *H*11), 2.33 (s, 3H, *H*19), (the hydrazone N–H proton was not observed due to severe broadening from rapid proton exchange); ^13^C NMR (126 MHz, CDCl_3_) *δ* 160.64, 159.38, 146.93, 138.43, 133.43, 129.31, 129.06, 128.20, 127.57, 125.68, 120.26, 99.77, 34.06, 16.24; ESI-MS (+) CH_3_OH *m*/*z* = 384 [TPP95 + H]^+^; Anal. calcd for C_23_H_21_N_5_O: C, 72.04; H, 5.52; N, 18.26%. Found: C, 72.01; H, 5.44; N, 18.20%.

**Scheme 9:**
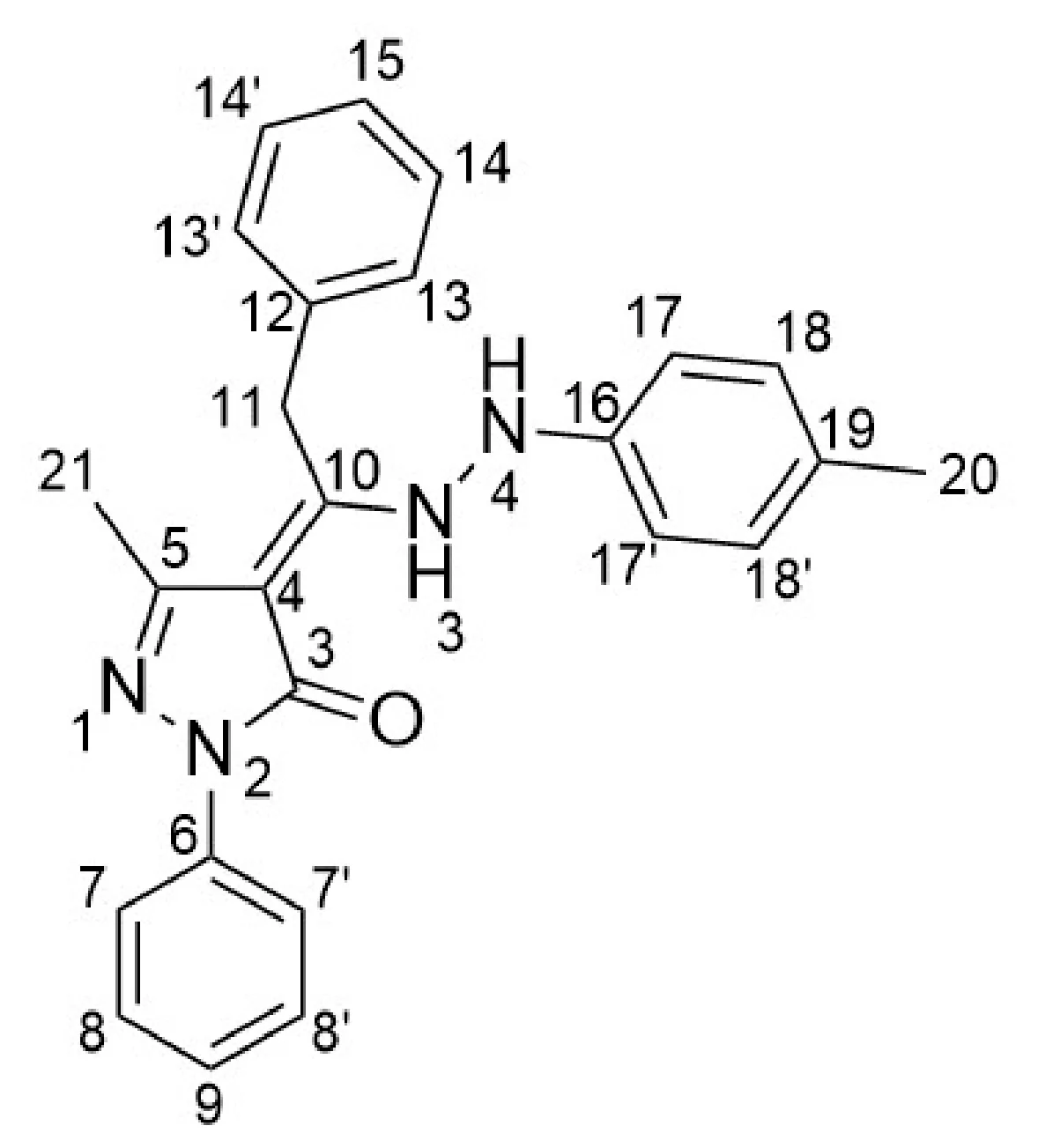
Compound TPP96.

Compound TPP96. (Scheme 9) (Z)-5-methyl-2-phenyl-4-(2-phenyl-1-(2-(*p*-tolyl)hydrazineyl)ethylidene)-2,4-dihydro-3*H*-pyrazol-3-one. TPP96 was synthesized from HQ^Ph,Bn^ (310 mg, 1.06 mmol) and 4-methylphenylhydrazine hydrochloride, following the general procedure previously described (reaction time 4 h). Yellow solid (377 mg, yield: 90%), mp 158–159 °C. ^1^H NMR (500 MHz, CDCl_3_) *δ* 7.34 (d, *J* = 7.5 Hz, 2H, *H*7,7′), 7.28 (m, 3H, *H*18,18′, *H15*), 7.17 (t, *J* = 7.2 Hz, 3H, *H*8,8′, *H*9), 7.16–7.04 (m, 4H, *H*14,14′, *H*13,13′), 7.00 (d, *J* = 7.9 Hz, 2H, *H*17,17′), 6.89 (brm, 2H, N3-*H*, N4-*H*), 4.08 (s, 2H, *H*11), 2.36 (s, 3H, *H*21), 1.83 (s, 3H, *H*20); ^13^C NMR (126 MHz, CDCl_3_) *δ* 164.36, 152.47, 140.61, 138.88, 138.55, 135.66, 129.92, 129.57, 129.32, 128.90, 128.69, 127.20, 126.70, 125.25, 112.30, 33.02, 21.26, 12.32; ESI-MS (+) CH_3_CN *m*/*z* = 397 [TPP96 + H]^+^; 419 [TPP96 + H + Na]^+^; 435 [TPP96 + H + K]^+^; Anal. calcd for C_25_H_24_N_4_O: C, 75.73; H, 6.10; N, 14.13%. Found: C, 75.67; H, 6.02; N, 14.07%.

### 4.3. Cell Lines

Human GBM cell lines U87 and T98 (European Collection of Cell Cultures, Salisbury, UK) were maintained in Eagle’s Minimum Essential Medium (EMEM; S.I.A.L., Rome, Italy) enriched with 10% (*v*/*v*) heat-inactivated fetal bovine serum (FBS), penicillin (100 IU/mL), streptomycin (100 μg/mL), 2 mmol/L L-glutamine, 10% (*v*/*v*) nonessential amino acids, and 10% (*v*/*v*) sodium pyruvate.

The non-tumoral primary human fibroblast cell line NHF A12 (kindly provided by IFO, Istituti Fisioterapici Ospitalieri, Rome, Italy [24]) was cultured in Dulbecco’s Modified Eagle’s Medium (DMEM; S.I.A.L.) supplemented with 10% (*v*/*v*) FBS, penicillin (100 IU/mL), and streptomycin (100 μg/mL). All cultures were incubated at 37 °C under humidified conditions with 5% CO_2_. All cell cultures were routinely tested for mycoplasma contamination using the PCR Mycoplasma Detection & Elimination kit (Applied Biological Materials Inc., Richmond, BC, Canada) and remained strictly negative throughout the study.

### 4.4. Cell Viability Assay

GBM and NHF A12 cells (3 × 10^4^ cells/mL) were seeded in 96-well plates, in a final volume of 100 μL/well, and allowed to adhere overnight. The optimal initial seeding density for each cell line was carefully selected based on preliminary optimization assays to ensure that the absorbance values remained strictly within the reliable, non-saturated range of the MTT response throughout the experimental timeframe. After one day of incubation, synthetic compounds were added. At least six replicates in each experiment were used for each treatment. The final concentration of DMSO in the culture medium never exceeded 0.05% (*v*/*v*) for all treatments and vehicle controls, showing no statistically significant differences compared to completely untreated cells. After 72 h, cell viability was assessed by adding 0.8 mg/mL of 3-(4,5-dimethylthiazol-2-yl)-2,5-diphenyltetrazolium bromide (MTT) (Sigma-Aldrich, Milan, Italy) to the media. The absorbance of samples, solubilized in dimethyl sulfoxide (DMSO), compared to vehicle-treated cells was measured at 570 nm using the SpectraMax iD3 microplate reader (Molecular Devices, München, Germany).

The Selectivity Index (SI) was calculated to determine the degree of tumor selectivity of the compound TPP25. It was defined as the ratio of the IC_50_ value of non-malignant cells to the IC_50_ value of cancer cells. Since the IC_50_ against normal cells (NHF A12) was not reached, the highest tested concentration (20 μM) was used as a conservative baseline to express the minimum SI value (SI > value).

### 4.5. Cell Cycle Assay

GBM cells (5 × 10^4^ cells/mL) seeded in 6-well plates were exposed to TPP25 for 72 h. Next, the harvested cells were fixed in cold 70% ethanol, treated for 30 min at 37 °C with 100 μg/mL ribonuclease A solution (Sigma-Aldrich), stained for 30 min at room temperature with propidium iodide (PI, Sigma-Aldrich) 20 μg/mL, and analyzed using a BD Accuri C6 Plus flow cytometer (BD Biosciences, Milan, Italy) and BD Accuri C6 Plus Software version 1.0.34.1, measuring the fluorescence emission on FL-2.

### 4.6. Cell Death Assay

GBM cells (5 × 10^4^ cells/mL) were treated with TPP25 for up to 72 h. At designated time points, cells were harvested, washed, and incubated with 2 μg/mL of PI for 10 min at room temperature in the dark. Fluorescence emission was analyzed using a BD Accuri C6 Plus flow cytometer and its dedicated software, measuring the signal in the FL-2 channel. To ensure data reliability, cellular debris was excluded from the analysis by gating on forward-scatter versus side-scatter (FSC vs. SSC) profiles. Furthermore, a strict doublet exclusion gating strategy was systematically applied based on PI fluorescence pulse area versus pulse height (PI-A vs. PI-H) parameters to quantify single-cell events only. Since PI was utilized as a single-color fluorochrome for this primary screening, spectral compensation was not required.

To comprehensively map the chronological induction and execution of apoptosis, a dual-staining assay was performed after 24, 48, and 72 h of treatment utilizing an Annexin V-FITC/7-AAD Apoptosis Detection Kit (BD Biosciences) according to the manufacturer’s instructions. Briefly, GBM cells were washed twice with ice-cold PBS and resuspended in 1× Binding Buffer at a final concentration of approximately 1 × 10^6^ cells/mL. Cells were then incubated with 5 μL of Annexin V-FITC and 5 μL of 7-aminoactinomycin D (7-AAD) solution for 15 min at room temperature in the dark. Samples were analyzed within 1 h on the BD Accuri C6 Plus flow cytometer. FITC fluorescence was captured in the FL-1 channel, and 7-AAD fluorescence was collected in the FL-3 channel. Cellular debris and doublets were excluded from the analysis. For this multi-color assay, software-based spectral compensation was applied using single-stained control cell samples to eliminate any fluorescence spillover from the FITC spectrum into the 7-AAD detection channel. Quadrant gating was utilized to differentiate cells into viable (Annexin V^−^/7-AAD^−^), early apoptotic (Annexin V^+^/7-AAD^−^), and late apoptotic/secondary necrotic (Annexin V^+^/7-AAD^+^) populations.

### 4.7. ROS Production

Intracellular oxidative stress levels in GBM cells were evaluated using the fluorescent probe DCFDA (Thermo Fisher Scientific, Segrate, Italy). Cells (5 × 10^4^ cells/mL) were treated with TPP25 for up to 72 h. H_2_O_2_ (0, 6%) was used as a positive control. At the end of each incubation period, cells were incubated with DCFDA (10 μM) at 37 °C for 20 min. The same samples were then analyzed to assess ROS production both quantitatively and qualitatively. Quantitative measurements were performed by flow cytometry using a BD Accuri C6 Plus system and related software, whereas qualitative evaluation was carried out by fluorescence microscopy using an Olympus IX70 microscope (Evident Europe GmbH, Hamburg, Germany) equipped with OLYMPUS cellSens Standard v4.3 software (Evident Europe GmbH).

### 4.8. Mitochondrial Transmembrane Potential

The ΔΨ_m_ was evaluated by 5,5′,6,6′-tetrachloro-1,1′,3,3′-tetraehylbenzimidazolylcarbocyanineiodide (JC1, Thermo Fisher Scientific) staining. GBM cell lines (5 × 10^4^ cells/mL) were seeded into 24-well plates, treated with TPP25 or vehicle for up to 72 h. Subsequently, cells were incubated with JC-1 (10 μg/mL). Carbonyl cyanide chlorophenylhydrazone protonophore (CCCP, 50 μM, Sigma-Aldrich), a mitochondrial uncoupler known to dissipate, was used as a positive control. Following staining, the same samples were used to evaluate mitochondrial membrane potential both quantitatively and qualitatively. Quantitative analysis was performed by flow cytometry using a BD Accuri C6 Plus system, while qualitative assessment was carried out by fluorescence microscopy using an Olympus IX70 microscope.

### 4.9. Western Blot

Lysates from GBM cells were extracted using a lysis buffer containing a protease-inhibitor cocktail (EuroClone, Milan, Italy). Proteins were separated on 12% SDS/polyacrylamide gels using a Mini-PROTEAN Tetra Cell system (Bio-Rad, Milan, Italy). Protein transfer to a nitrocellulose membrane was performed using a Mini Trans-Blot Turbo RTA system (Bio-Rad). To assess total protein loading and transfer efficiency, membranes were stained with Ponceau S solution (Sigma-Aldrich) and digitally imaged prior to blocking. Non-specific binding sites were blocked with low-fat dry milk or BSA in PBS containing 0.1% Tween 20. Membranes were incubated with phospho-histone H2AX (#9718, 1:1000; Cell Signaling Technology, Milan, Italy), Cleaved Caspase-3 (#96647, 1:1000; Cell Signaling Technology), GAPDH (sc-47724, 1:1000; Santa Cruz Biotechnology, Segrate, Italy), β-actin (#4967, 1:1000; Cell Signaling Technology) antibodies overnight at 4 °C, followed by incubation with HRP-conjugated secondary antibodies (anti-rabbit Ab, 1:2000; anti-mouse, 1:2000; Cell Signaling Technology). Chemiluminescent signals were detected using LiteAblot PLUS or Turbo kits (EuroClone) on a ChemiDocTM XRS+ system (Bio-Rad). Densitometric analysis of the bands was performed using Image Lab software (version 6.1, Bio-Rad) with uniform background subtraction applied across all lanes via a rolling disc algorithm. GAPDH was employed as the primary loading control, while β-actin and total protein staining (Ponceau S) were used as alternative normalization references to cross-validate data quantification under high-dose cytotoxic conditions. For each target protein, densitometric quantification was calculated from three independent replicates (*n* = 3).

### 4.10. Colony Formation Assay

GBM cells were seeded in six-well plates at a density of 500 cells per well and allowed to adhere overnight. Cells were then treated with TPP25 or vehicle for 14 days, with medium replacement every 3 days. After 2 weeks, colonies were fixed with 4% paraformaldehyde for 15 min, washed with PBS, and stained with 0.1% crystal violet (Sigma-Aldrich) for 15 min. Colonies were visualized and counted under a microscope. To evaluate clonogenic survival, the raw colony counts were normalized and expressed as the percentage of clone formation relative to the untreated control group. Additionally, the total percentage of surface area occupied by the colonies in each well was quantified using ImageJ software (version 1.54 g).

### 4.11. Statistical Analysis

GraphPad Prism 9.0.0(121) software (GraphPad Software, San Diego, CA, USA) was used for statistical analysis. IC_50_ values and their corresponding 95% confidence intervals (95% CI) were determined via non-linear regression analysis utilizing the profile likelihood method. The biological assay results represent the mean ± Standard Error of the Mean (SEM) of three experiments. Data were first verified for their normal Gaussian distribution by means of the Shapiro–Wilk normality test. Normally distributed data were analyzed by one-way or two-way analysis of variance (ANOVA) followed by Tukey’s or Dunnett’s multiple comparison tests, where appropriate (* *p* < 0.05, ** *p* < 0.01, *** *p* < 0.001, **** *p* < 0.0001). Specifically, one-way ANOVA with Tukey’s post hoc test was applied for all-to-all pairwise comparisons of a single variable, while Dunnett’s test was chosen to compare multiple groups strictly against the untreated control. Two-way ANOVA followed by Tukey’s test was utilized for multi-variable experimental designs to evaluate the concurrent effects and interactions of both time and treatment factors. The final concentration of DMSO in the culture medium never exceeded 0.05% (*v*/*v*) for all treatments and vehicle controls, showing no statistically significant differences compared to completely untreated cells.

## 5. Conclusions

The treatment of GBM still represents one of the main oncology challenges due to its aggressive nature and resistance to conventional therapies. The poor prognosis for GBM patients results in high demand for the discovery of novel therapeutic agents. For these reasons, in the present study, a novel series of pyrazolone-based hydrazones was synthesized and evaluated in vitro. Among the series, TPP25 emerged as the most interesting lead compound for further preclinical optimization.

In this regard, a Structure–Activity Relationship study was conducted, revealing that the –CF_3_ group was a key structural determinant for the biological activity. Our specific experimental findings demonstrate that TPP25 exhibits robust in vitro cytotoxic efficacy against both U87 and T98 GBM cell lines. Mechanistically, the compound triggers a coordinated cascade of intracellular events, characterized by reactive oxygen species production and mitochondrial membrane depolarization. This bioenergetic collapse drives severe genotoxic stress and culminates in cell death. Crucially, our findings support line-specific response profiles during late-stage cell death. At prolonged exposure, T98 cells display a cumulative genotoxic stress with a dramatic accumulation of γ-H2AX, whereas U87 cells undergo a decrease in γ-H2AX signal reflecting a faster transition into an advanced, irreversible phase of cell death. Ultimately, this mechanism translates into a permanent, long-term suppression of GBM colony-forming ability, effectively preventing cell self-renewal. While TPP25 preserved the viability of non-tumoral NHF A12 fibroblasts, evaluating selectivity against a single cell type represents only an initial screening step and is insufficient to prove overall biocompatibility.

Although these findings provide a valuable initial proof-of-concept for the antitumor potential of TPP25, the compound should not be considered a ready-made therapeutic agent. Additional studies will be required to optimize the chemical scaffold, improve its pharmacological properties, and map its safety profile.

Future investigations should evaluate whether mitochondrial dysfunction represents the primary molecular target or a downstream consequence of oxidative stress, and extend the toxicity profiles to specialized non-tumoral models such as normal astrocytes. Finally, given the intrinsic challenge represented by drug delivery to the central nervous system, dedicated blood–brain barrier penetration assays are required. The incorporation of TPP25 into advanced brain-targeted delivery systems may represent a future strategy to enhance brain accumulation, improve tumor selectivity, and minimize systemic toxicity.

## Figures and Tables

**Figure 2:**
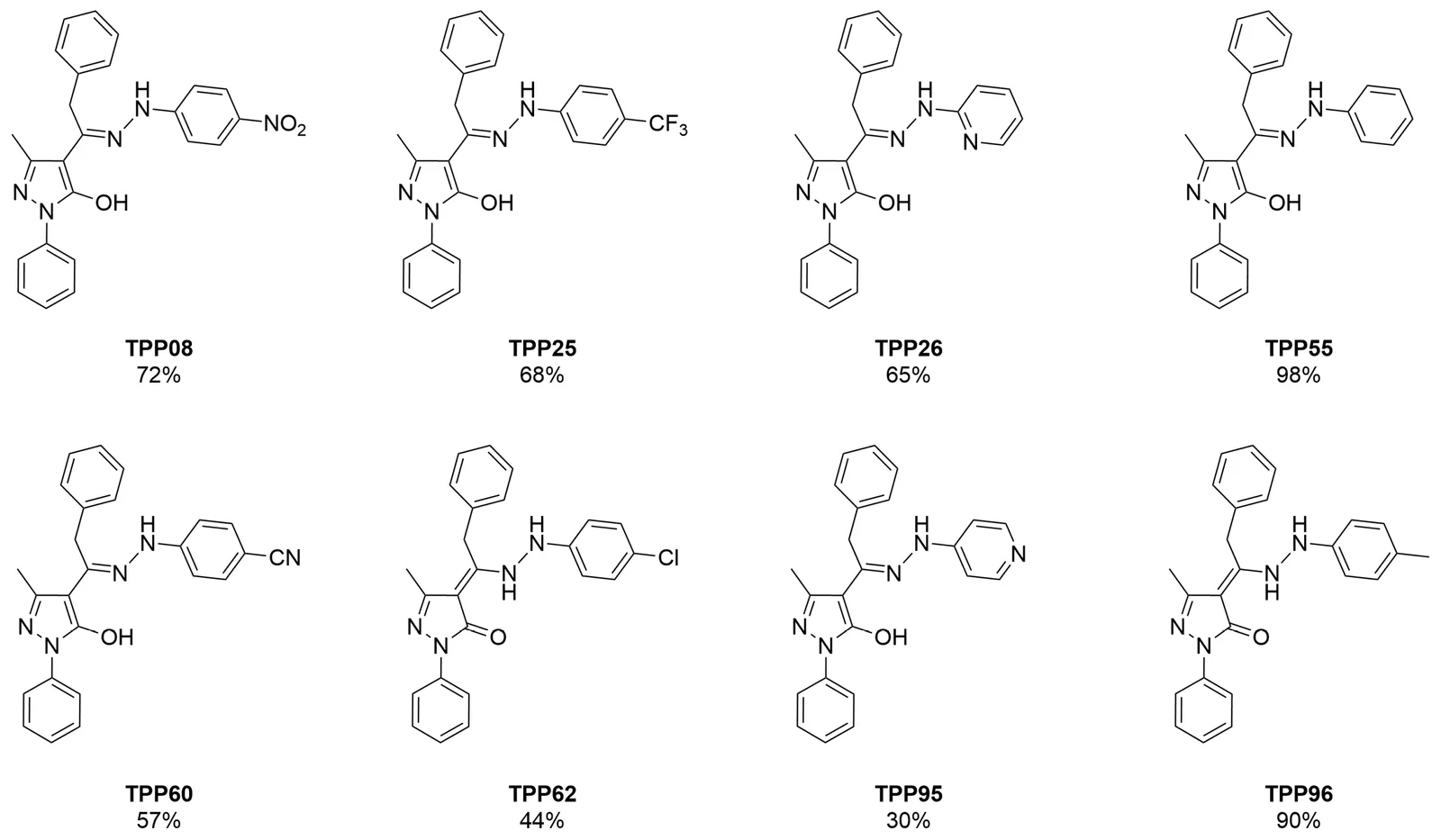
Synthesized TPP compounds.

**Table 1:** Cytotoxic activity of TPP series in T98 and U87 GBM cell lines.

Compound	T98		U87	
	IC_50_ ^a^	95% CI ^b^	IC_50_ ^a^	95% CI ^b^
TPP08	—	—	—	—
TPP25	11.01	10.42 to 11.64	13.12	10.23 to 16.87
TPP26	—	—	—	—
TPP55	28.97	14.35 to 58.48	23.07	14.26 to 37.41
TPP60	—	—	49.32	24.21 to 100.23
TPP62	—	—	—	—
TPP95	—	—	—	—
TPP96	—	—	—	—
TMZ ^c^	—	—	—	—

^a^ IC_50_ values in μM as mean of *n* = 3 independent biological experiments. Each independent determination was conducted with at least six technical replicates. ^b^ In μM, 95% Confidence Intervals (profile likelihood) determined from the non-linear regression analysis. ^c^ IC_50_ values of temozolomide (TMZ) previously reported [16]. — The maximum inhibitory effect was not reached up to the highest concentration tested (50 μM); therefore, the IC_50_ is estimated to be greater than 50 μM (>50 μM), or there is a lack of cytotoxicity within the tested concentration range or partial cytotoxicity observed, but data points could not be reliably fitted to a sigmoidal model (Figures S17 and S18).

## Data Availability

The original contributions presented in this study are included in the article and Supplementary Materials. Further inquiries can be directed to the corresponding authors.

## Supplementary Materials

The following supporting information can be downloaded at: https://www.mdpi.com/article/10.3390/ph19091335/s1, Figure S1: ^1^H NMR Spectrum (500 MHz, CDCl_3_) of Compound TPP08; Figure S2: ^13^C NMR Spectrum (126 MHz, CDCl_3_) of Compound TPP08; Figure S3: ^1^H NMR Spectrum (500 MHz, CDCl_3_) of Compound TPP25; Figure S4: ^13^C NMR Spectrum (126 MHz, CDCl_3_) of Compound TPP25; Figure S5: ^1^H NMR Spectrum (500 MHz, CDCl_3_) of Compound TPP26; Figure S6: ^13^C NMR Spectrum (126 MHz, CDCl_3_) of Compound TPP26; Figure S7: ^1^H NMR Spectrum (500 MHz, CDCl_3_) of Compound TPP55; Figure S8: ^13^C NMR Spectrum (126 MHz, CDCl_3_) of Compound TPP55; Figure S9: ^1^H NMR Spectrum (500 MHz, CDCl_3_) of Compound TPP60; Figure S10: ^13^C NMR Spectrum (126 MHz, CDCl_3_) of Compound TPP60; Figure S11: ^1^H NMR Spectrum (500 MHz, CDCl_3_) of Compound TPP62; Figure S12: ^13^C NMR Spectrum (126 MHz, CDCl_3_) of Compound TPP62; Figure S13: ^1^H NMR Spectrum (500 MHz, CDCl_3_) of Compound TPP95; Figure S14: ^13^C NMR Spectrum (126 MHz, CDCl_3_) of Compound TPP95; Figure S15: ^1^H NMR Spectrum (500 MHz, CDCl_3_) of Compound TPP96; Figure S16: ^13^C NMR Spectrum (126 MHz, CDCl_3_) of Compound TPP96; Figure S17: Cytotoxic effect of TPP compounds on T98 GBM cell line; Figure S18: Cytotoxic effect of TPP compounds on U87 GBM cell line; Figure S19: Annexin V/7-AAD-based assessment of TPP25-induced cell death in GBM cell lines.

## Abbreviations

The following abbreviations are used in this manuscript:

GBM	Glioblastoma
IDH1/2	Isocitrate dehydrogenase 1 or 2
MGMT	O^6^-methylguanine-DNA methyltransferase
TMZ	Temozolomide
TPPs	Pyrazolone-based hydrazone compounds
HQ^Ph,Bn^	1-(5-hydroxy-3-methyl-1-phenyl-1*H*-pyrazol-4-yl)-2-phenylethan-1-one
h	hours
IC_50_	Half-maximal inhibitory concentration
MTT	3-(4,5-dimethylthiazol-2-yl)-2,5-diphenyltetrazolium bromide
n.c.	Not cytotoxic
n.f.	Not fittable
SEM	Standard Error of the Mean
IC_25_	Concentration that leaves 75% of cells viable (i.e., only 25% growth inhibition).
ɣ-H2AX	Phosphorylated histone H2AX
DSBs	Double-strand breaks
GAPDH	Glyceraldehyde 3-phosphate dehydrogenase
PI	Propidium iodide
MFI	Mean Fluorescence Intensity
ROS	Reactive oxygen species
DCFDA	2′,7′-dichlorodihydrofluorescein diacetate
DCF	2′,7′-dichlorofluorescein
H_2_O_2_	Hydrogen peroxide
ΔΨ_m_	Mitochondrial membrane potential
CCCP	Carbonyl cyanide m-chlorophenyl hydrazone
EMEM	Eagle’s Minimum Essential Medium
FBS	Fetal bovine serum
DMEM	Dulbecco’s Modified Eagle’s Medium
DMSO	Dimethyl sulfoxide
JC1	5,5′,6,6′-tetrachloro-1,1′,3,3′-tetraehylbenzimidazolylcarbocyanineiodide
