# Supplementary material for: Evaluation of Pyrazolone-Based Hydrazones as Potential Therapeutic Agents Against Glioblastoma
Source: Pharmaceuticals (Basel). 2026 Aug 24;19(9):1335. doi: 10.3390/ph19091335 (PMC13610654; doi:10.3390/ph19091335)
Supplement: Supplementary file 1 [file pharmaceuticals-19-01335-s001.zip › pharmaceuticals-4442079-supplementary.pdf]

Supplementary Materials

Evaluation of Pyrazolone-Based Hydrazones as Potential Therapeutic Agents Against Glioblastoma

Giorgio Cameli[1‡], Alessia Piergentili[2‡], Eleonora Spinozzi[2], Alessia Tombesi[2], Riccardo Petrelli[2*], Loredana Cappellacci[2], Maria Beatrice Morelli[1*]

[1] School of Pharmacy, Immunopathology and Molecular Medicine Unit, University of Camerino, Via Madonna delle Carceri 9, 62032 Camerino, Italy; giorgio.cameli@unicam.it (G.C.); mariabeatrice.morelli@unicam.it (M.B.M.)

[2] Chemistry Interdisciplinary Project (ChIP) research center, School of Pharmacy, University of Camerino, Via Madonna delle Carceri, 62032 Camerino, Italy; alessia.piergentili@unicam.it (A.P.); eleonora.spinozzi@unicam.it (E.S.); riccardo.petrelli@unicam.it (R.P.); loredana.cappellacci@unicam.it (L.C.)

[‡] These authors contributed equally

[*] Correspondence: riccardo.petrelli@unicam.it (R.P.); mariabeatrice.morelli@unicam.it (M.B.M.)

Table of Contents	Page
Figure S1 ^{1}H NMR Spectrum (500 MHz, $CDCl_3$) of Compound TPP08	2
Figure S2 ^{13}C NMR Spectrum (126 MHz, $CDCl_3$) of Compound TPP08	2
Figure S3 ^{1}H NMR Spectrum (500 MHz, $CDCl_3$) of Compound TPP25	3
Figure S4 ^{13}C NMR Spectrum (126 MHz, $CDCl_3$) of Compound TPP25	3
Figure S5 ^{1}H NMR Spectrum (500 MHz, $CDCl_3$) of Compound TPP26	4
Figure S6 ^{13}C NMR Spectrum (126 MHz, $CDCl_3$) of Compound TPP26	4
Figure S7 ^{1}H NMR Spectrum (500 MHz, $CDCl_3$) of Compound TPP55	5
Figure S8 ^{13}C NMR Spectrum (126 MHz, $CDCl_3$) of Compound TPP55	5
Figure S9 ^{1}H NMR Spectrum (500 MHz, $CDCl_3$) of Compound TPP60	6
Figure S10 ^{13}C NMR Spectrum (126 MHz, $CDCl_3$) of Compound TPP60	6
Figure S11 ^{1}H NMR Spectrum (500 MHz, $CDCl_3$) of Compound TPP62	7
Figure S12 ^{13}C NMR Spectrum (126 MHz, $CDCl_3$) of Compound TPP62	7
Figure S13 ^{1}H NMR Spectrum (500 MHz, $CDCl_3$) of Compound TPP95	8
Figure S14 ^{13}C NMR Spectrum (126 MHz, $CDCl_3$) of Compound TPP95	8
Figure S15 ^{1}H NMR Spectrum (500 MHz, $CDCl_3$) of Compound TPP96	9
Figure S16 ^{13}C NMR Spectrum (126 MHz, $CDCl_3$) of Compound TPP96	9
Figure S17 Cytotoxic effect of TPP compounds on T98 GBM cell line	10
Figure S18 Cytotoxic effect of TPP compounds on U87 GBM cell line	11
Figure S19 Annexin V/7-AAD-based assessment of TPP25-induced cell death in GBM cell lines	12

^{1}H NMR and ^{13}C NMR spectra

3-methyl-4-((E)-1-(2-(4-nitrophenyl)hydrazineylidene)-2-phenylethyl)-1-phenyl-1*H*-pyrazol-5-ol (Compound TPP08)

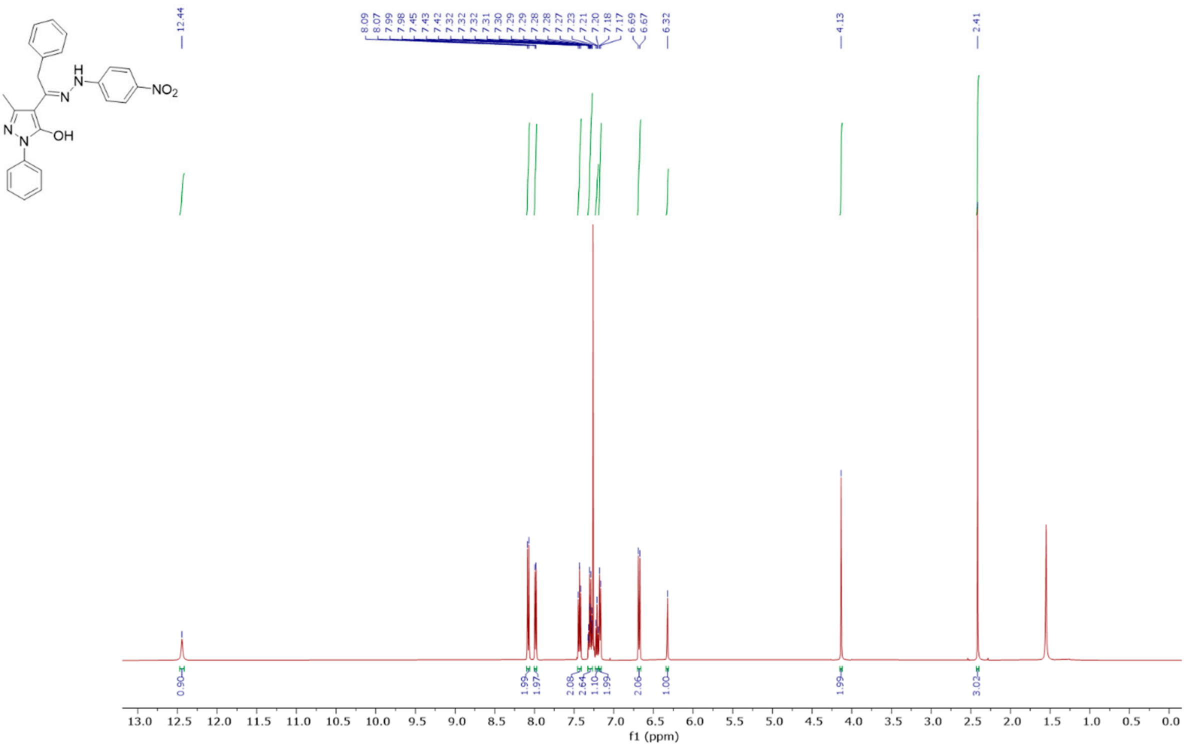

Figure S1. ^{1}H NMR Spectrum (500 MHz, $CDCl_3$) of Compound TPP08.

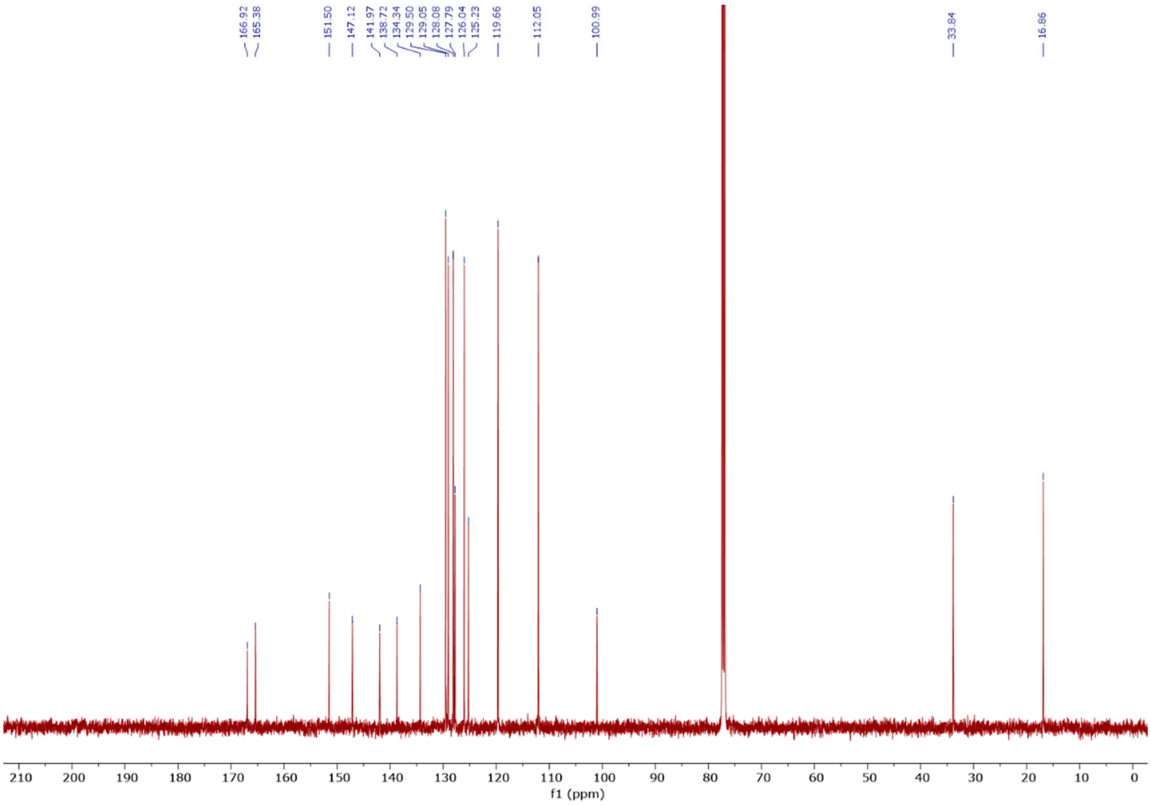

Figure S2. ^{13}C NMR Spectrum (126 MHz, $CDCl_3$) of Compound TPP08.

3-methyl-1-phenyl-4-((E)-2-phenyl-1-(2-(4-(trifluoromethyl)phenyl)hydrazineylidene)ethyl)-1*H*-pyrazol-5-ol (Compound TPP25).

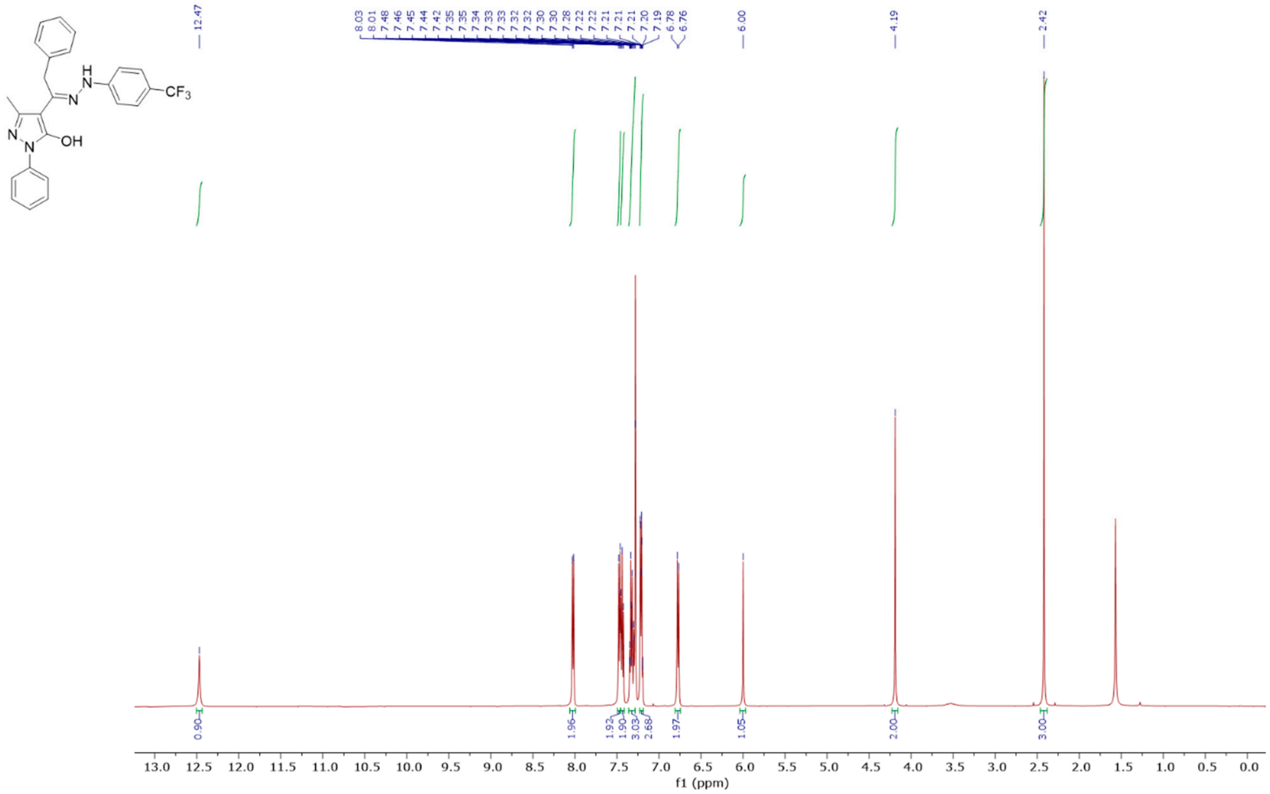

Figure S3. ^{1}H NMR Spectrum (500 MHz, $CDCl_3$) of Compound TPP25.

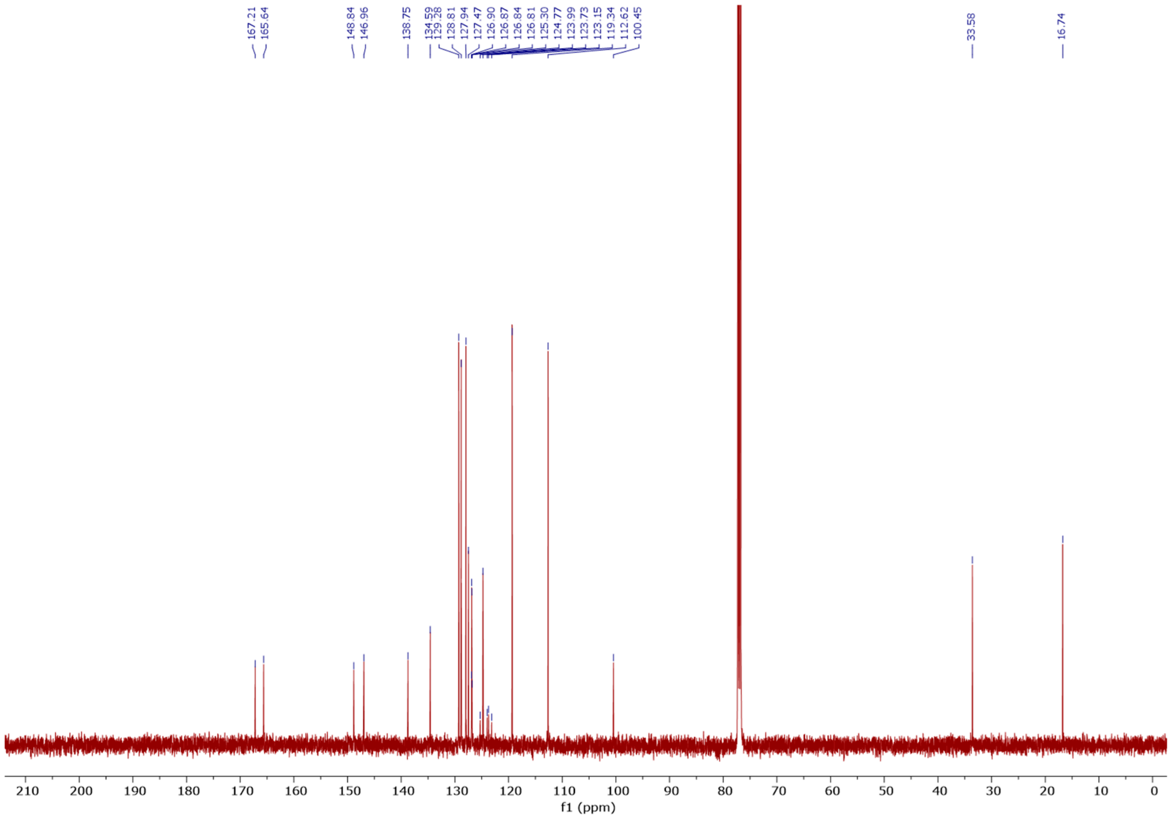

Figure S4. ^{13}C NMR Spectrum (126 MHz, $CDCl_3$) of Compound TPP25.

3-methyl-1-phenyl-4-((Z)-2-phenyl-1-(2-(pyridin-2-yl)hydrazineylidene)ethyl)-1*H*-pyrazol-5-ol (Compound TPP26).

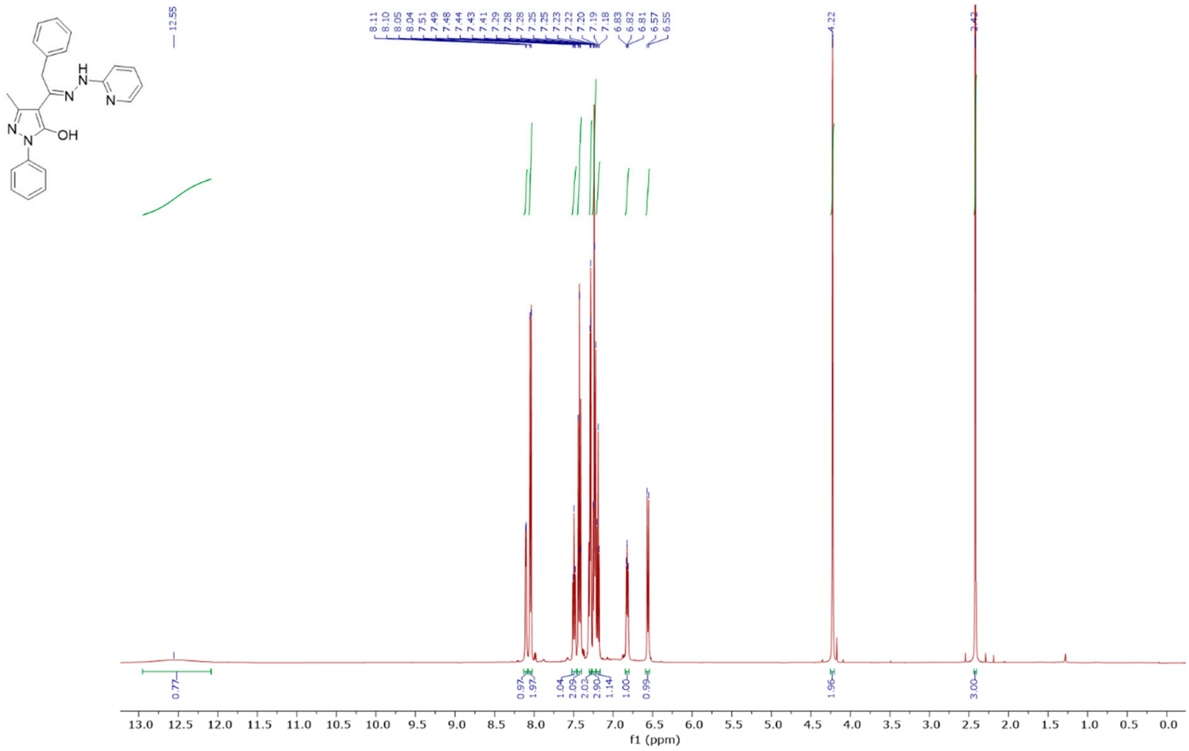

Figure S5. ^{1}H NMR Spectrum (500 MHz, $CDCl_3$) of Compound TPP26.

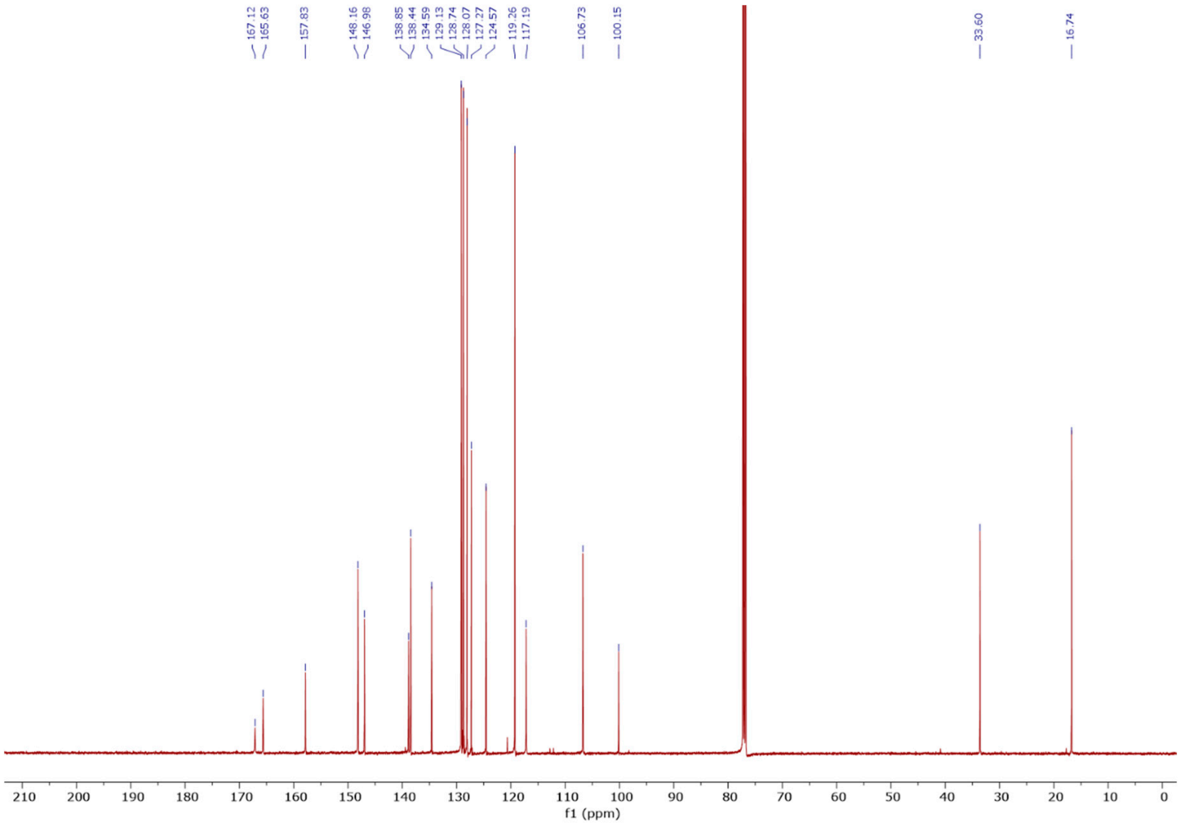

Figure S6. ^{13}C NMR Spectrum (126 MHz, $CDCl_3$) of Compound TPP26.

3-methyl-1-phenyl-4-((E)-2-phenyl-1-(2-phenylhydrazineylidene)ethyl)-1*H*-pyrazol-5-ol (Compound TPP55).

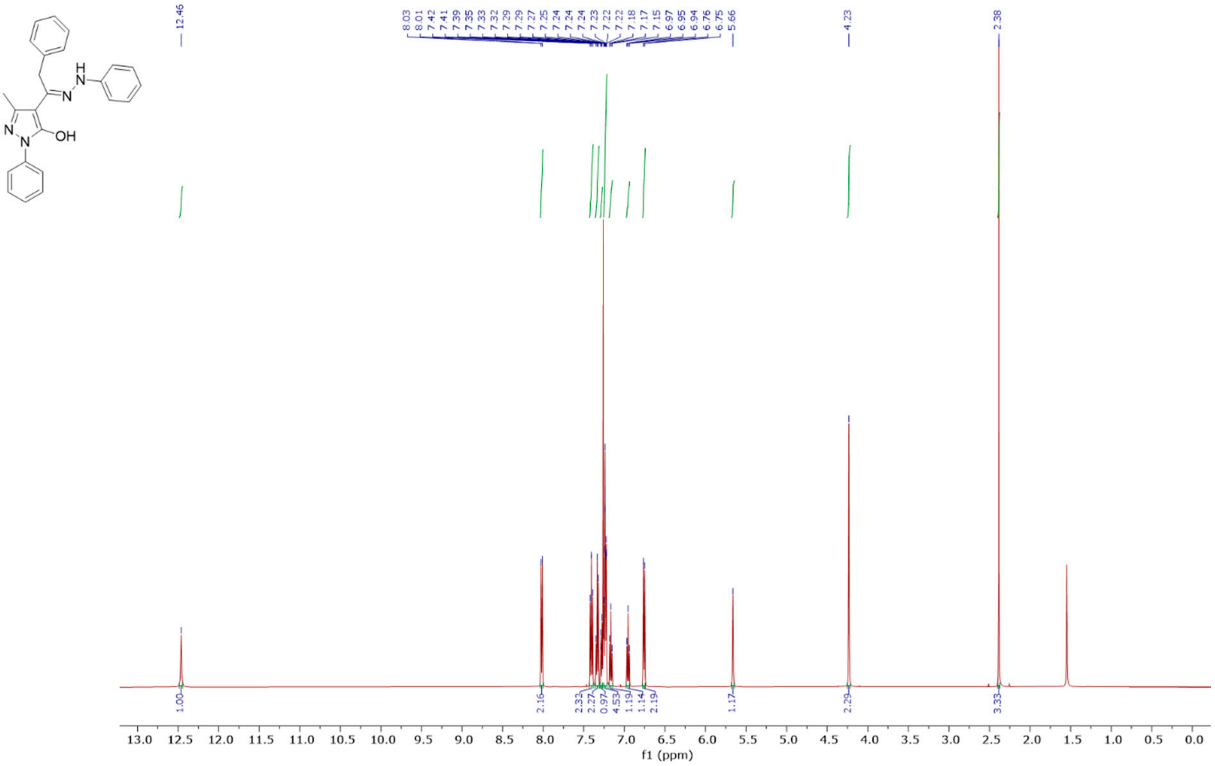

Figure S7. ^{1}H NMR Spectrum (500 MHz, $CDCl_3$) of Compound TPP55.

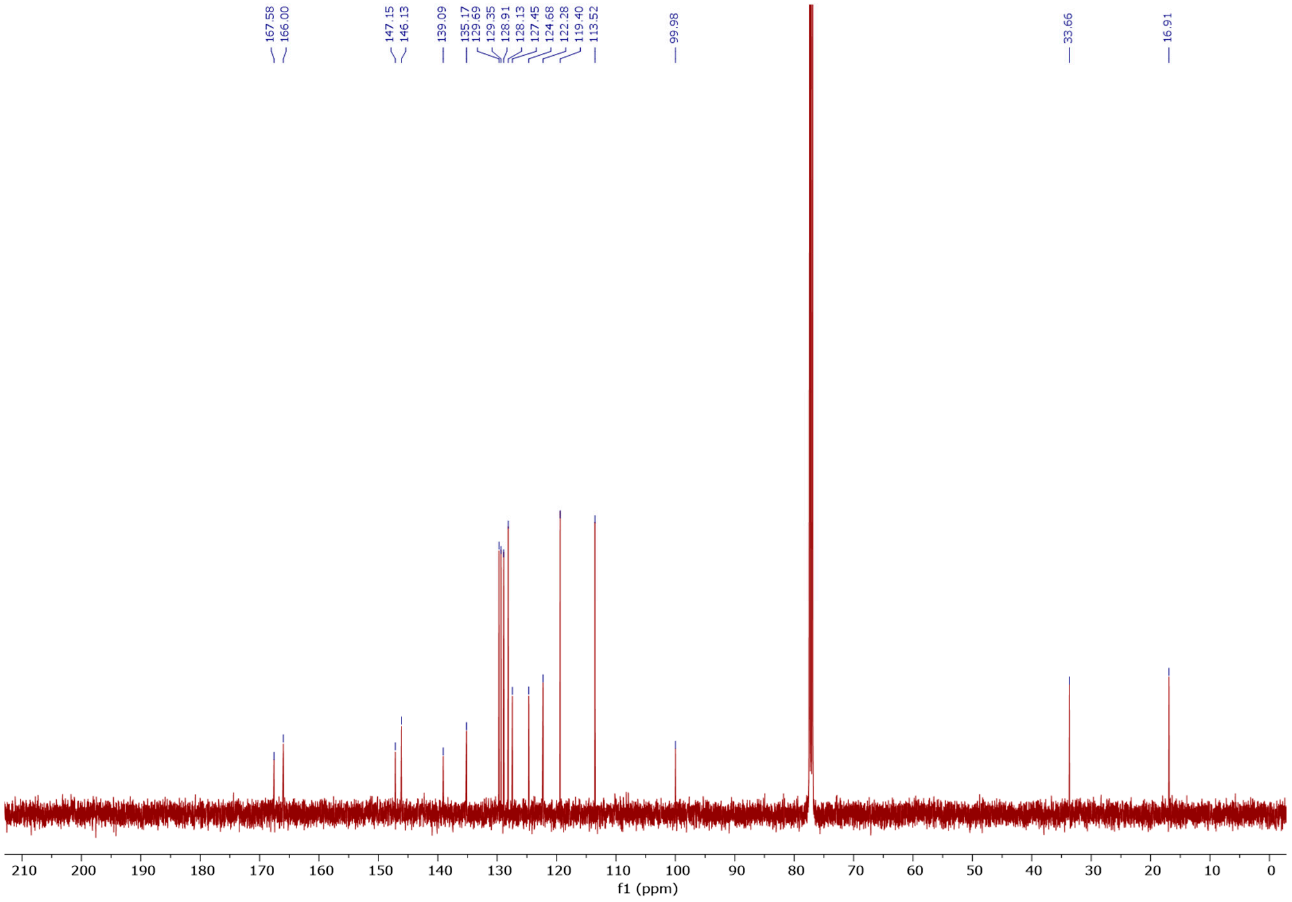

Figure S8. ^{13}C NMR Spectrum (126 MHz, $CDCl_3$) of Compound TPP55.

3-methyl-1-phenyl-4-((E)-2-phenyl-1-(2-(4-(cyano)phenyl)hydrazineylidene)ethyl)-1*H*-pyrazol-5-ol (Compound TPP60).

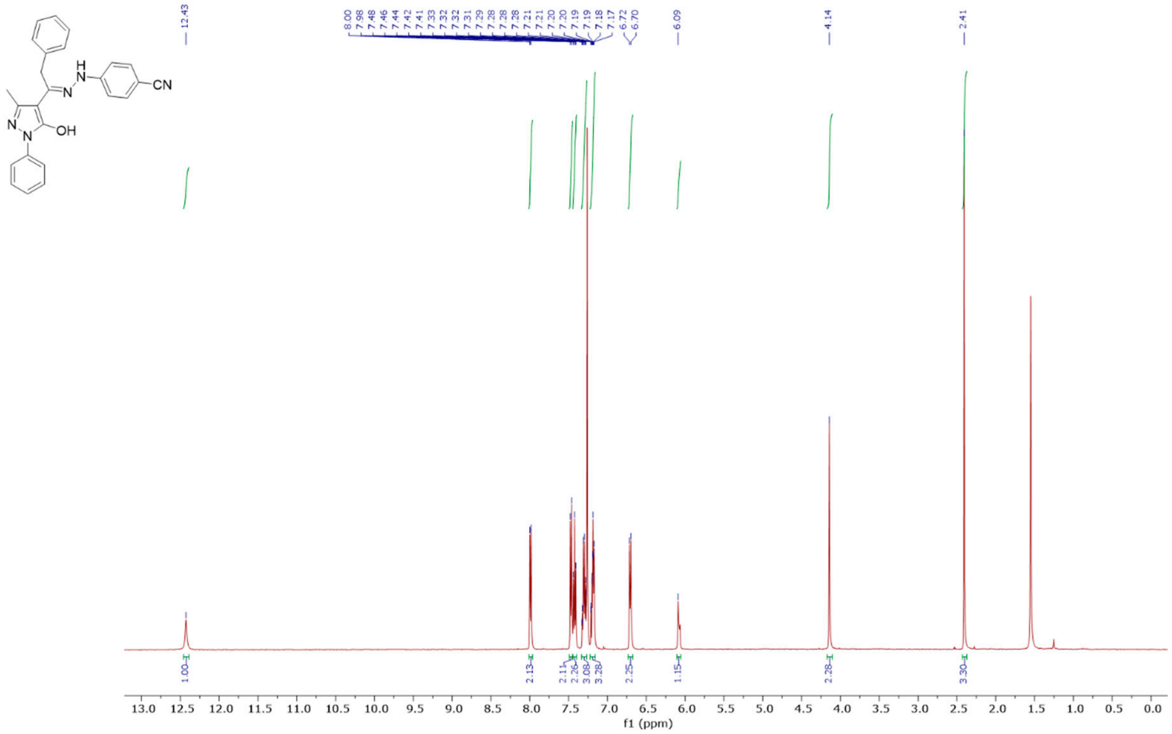

Figure S9. ^{1}H NMR Spectrum (500 MHz, $CDCl_3$) of Compound TPP60.

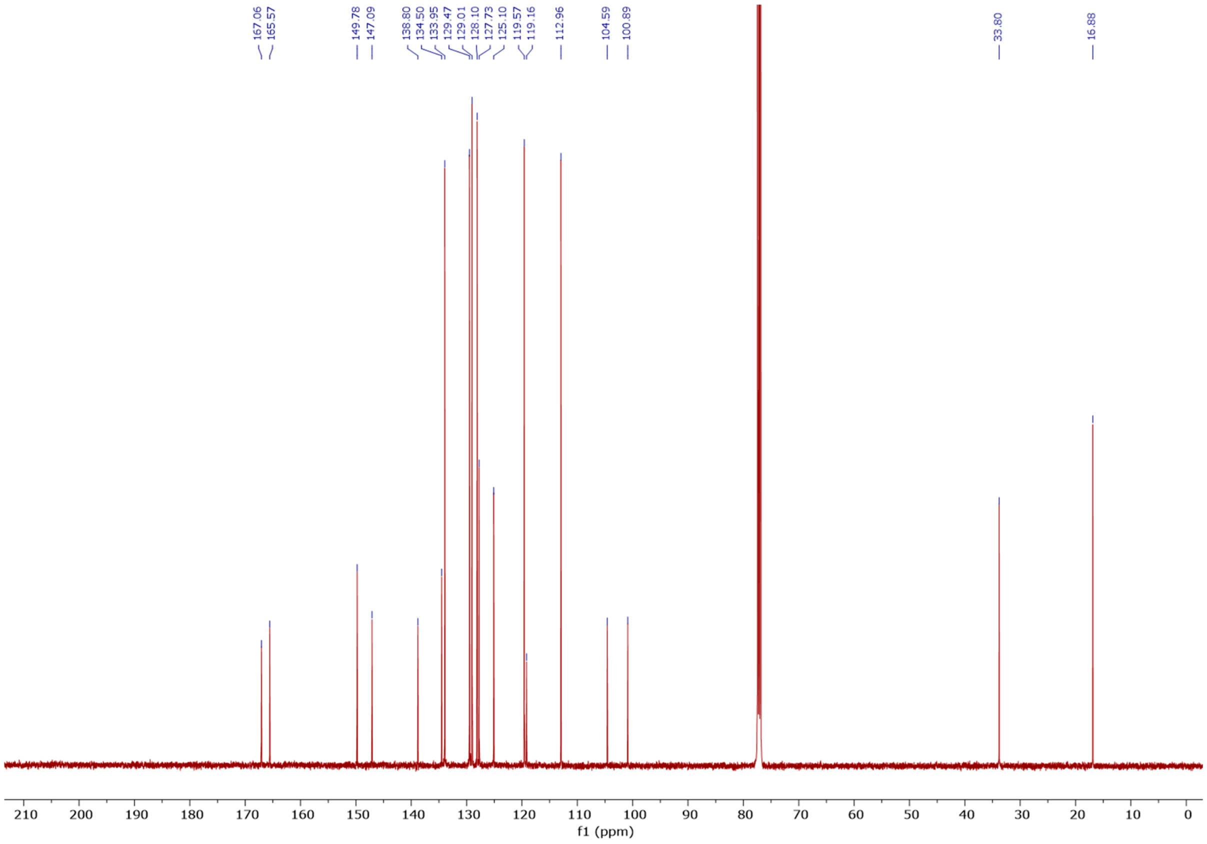

Figure S10. ^{13}C NMR Spectrum (126 MHz, $CDCl_3$) of Compound TPP60.

(Z)-4-(1-(2-(4-chlorophenyl)hydrazineyl)-2-phenylethylidene)-5-methyl-2-phenyl-2,4-dihydro-3*H*-pyrazol-3-one (Compound TPP62).

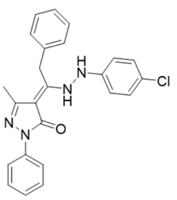

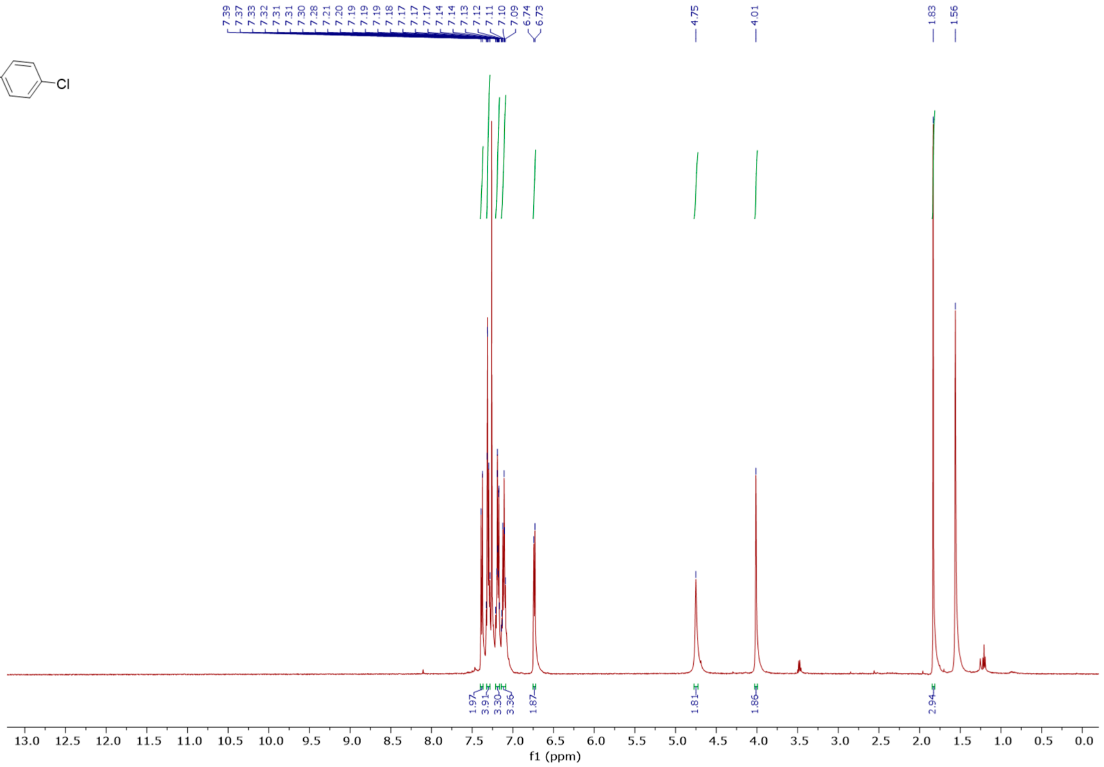

Figure S11. ^{1}H NMR Spectrum (500 MHz, $CDCl_3$) of Compound TPP62.

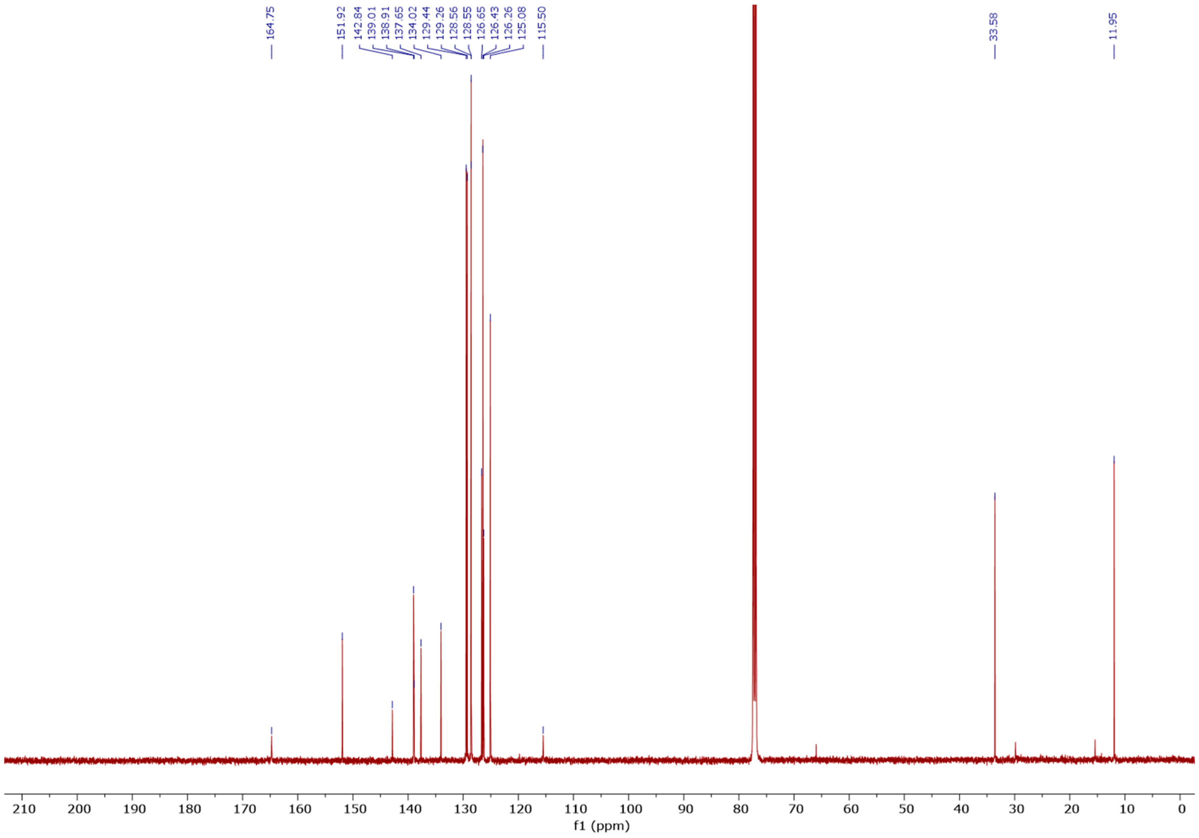

Figure S12. ^{13}C NMR Spectrum (126 MHz, $CDCl_3$) of Compound TPP62.

3-methyl-1-phenyl-4-((E)-2-phenyl-1-(2-(pyridin-4-yl)hydrazineylidene)ethyl)-1*H*-pyrazol-5-ol (Compound TPP95).

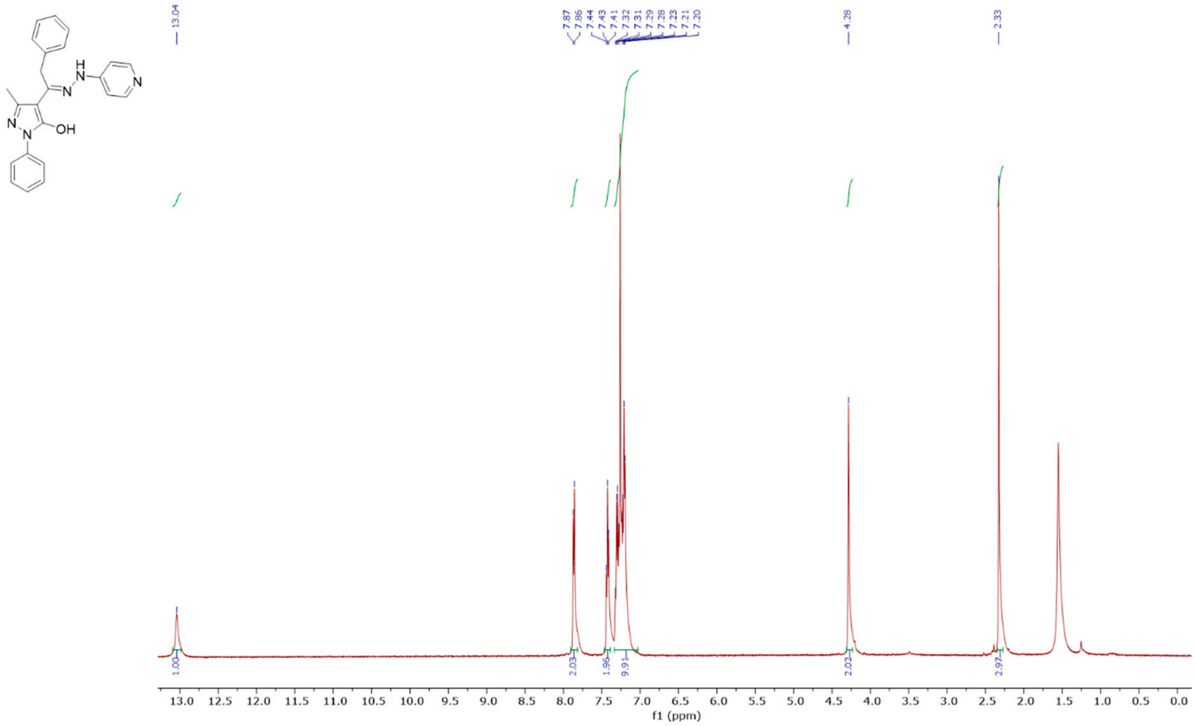

Figure S13. ^{1}H NMR Spectrum (500 MHz, $CDCl_3$) of Compound TPP95.

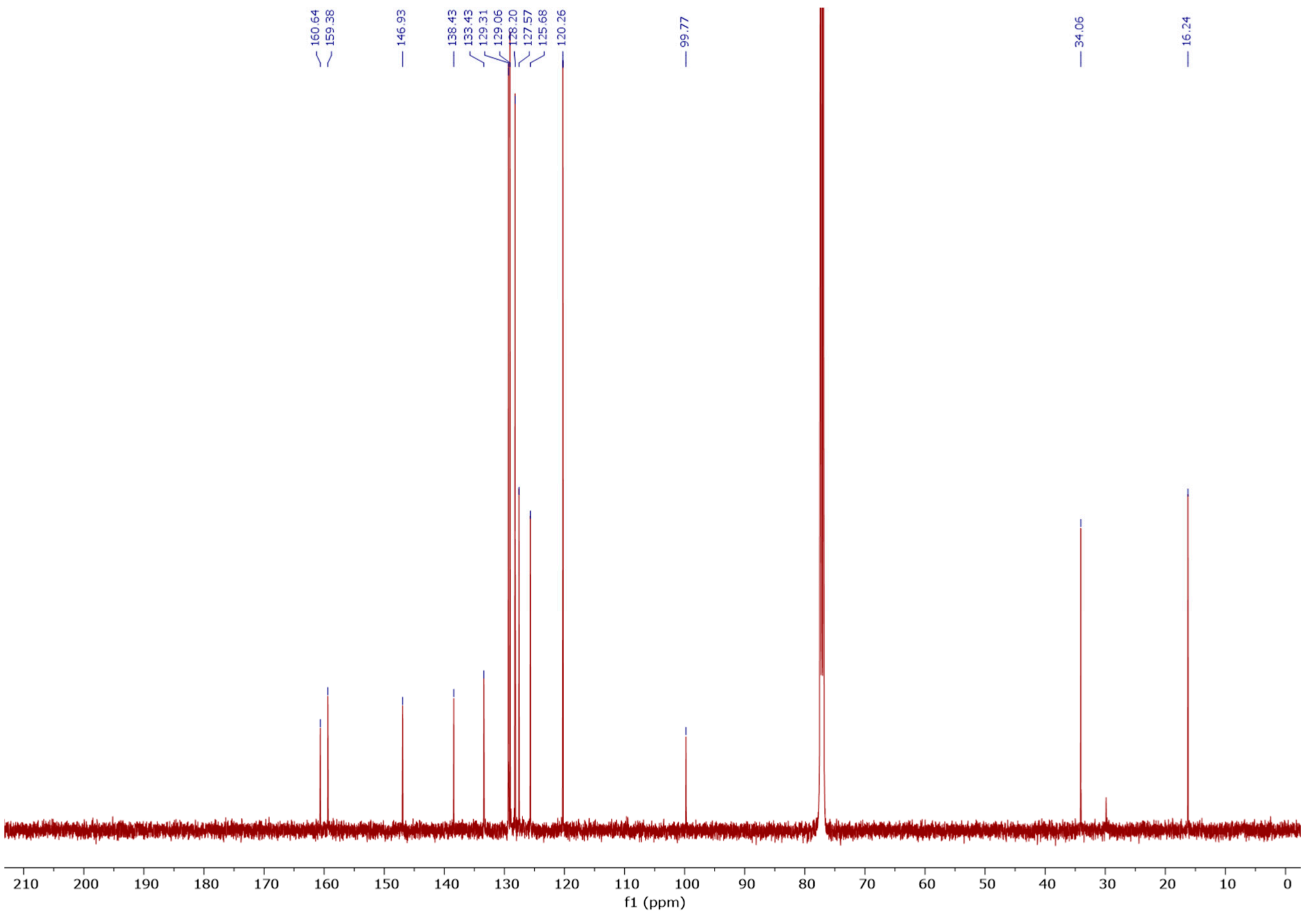

Figure S14. ^{13}C NMR Spectrum (126 MHz, $CDCl_3$) of Compound TPP95.

(Z)-5-methyl-2-phenyl-4-(2-phenyl-1-(2-(*p*-tolyl)hydrazineyl)ethylidene)-2,4-dihydro-3*H*-pyrazol-3-one (Compound TPP96).

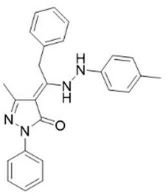

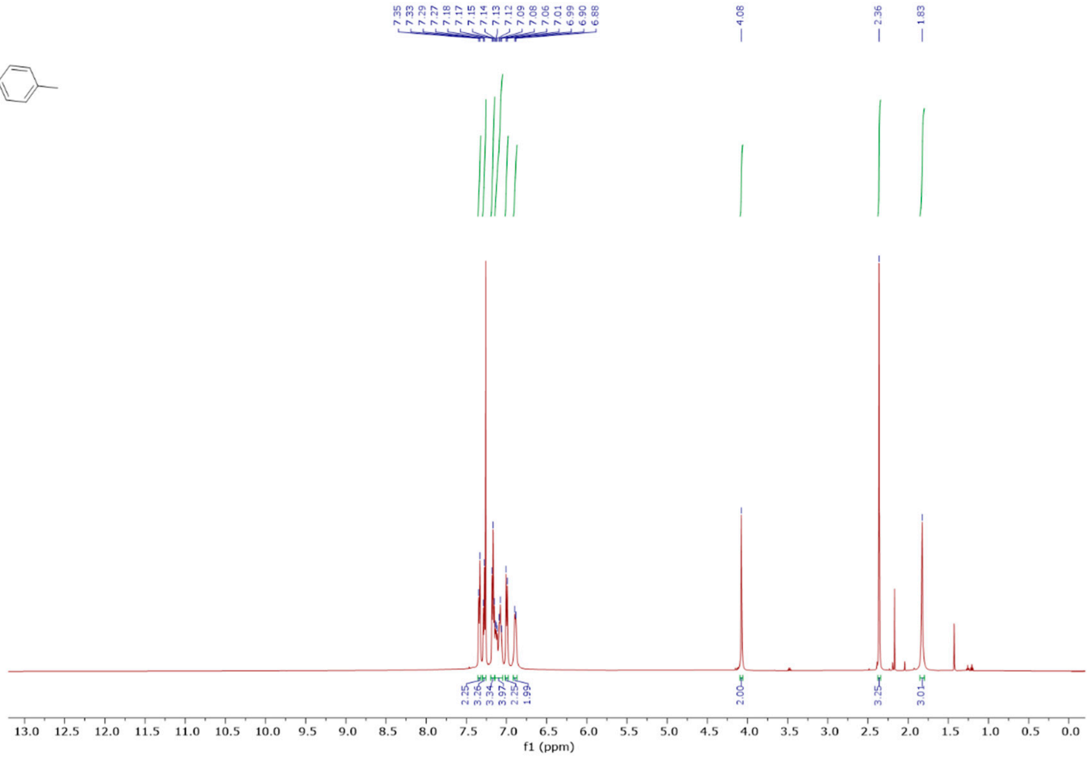

Figure S15. ^{1}H NMR Spectrum (500 MHz, $CDCl_3$) of Compound TPP96.

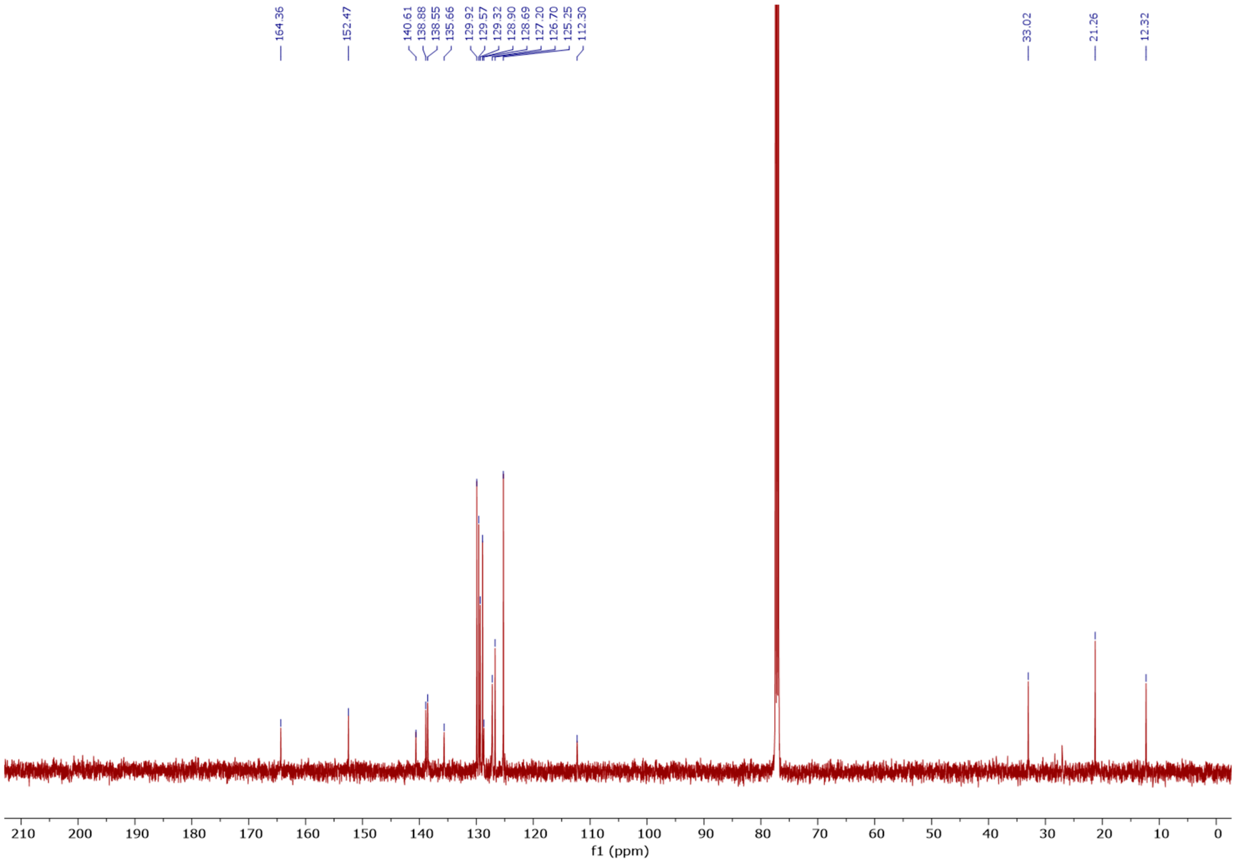

Figure S16. ^{13}C NMR Spectrum (126 MHz, $CDCl_3$) of Compound TPP96.

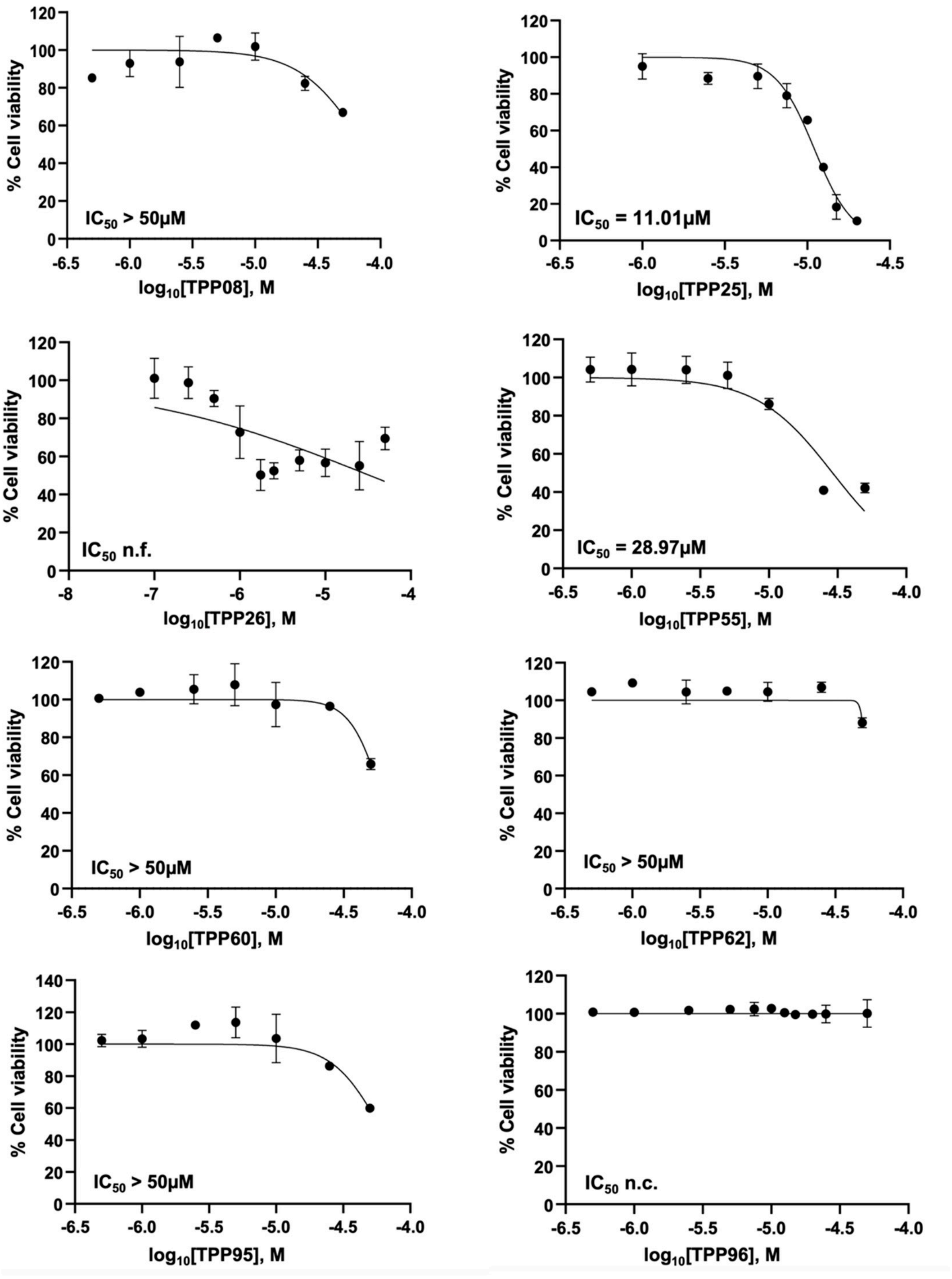

Figure S17. Cytotoxic effect of TPP compounds on T98 GBM cell line. Dose–response curves showing the percentage of cell viability (evaluated by MTT assay) as a function of the logarithmic concentration of the different compounds after 72 h of treatment. Concentrations on the x-axis are expressed as the base-10 logarithm of the molar concentration (log_{10}[TPP25], M). The respective IC_{50} values (half-maximal inhibitory concentration) are indicated for each compound. For curves where a standard IC_{50} could not be calculated: n.c. (not cytotoxic) indicates a lack of cytotoxicity within the tested concentration range; n.f. (not fittable) indicates an incomplete or non-sigmoidal cytotoxic profile where data could not be fitted to a standard sigmoidal model.

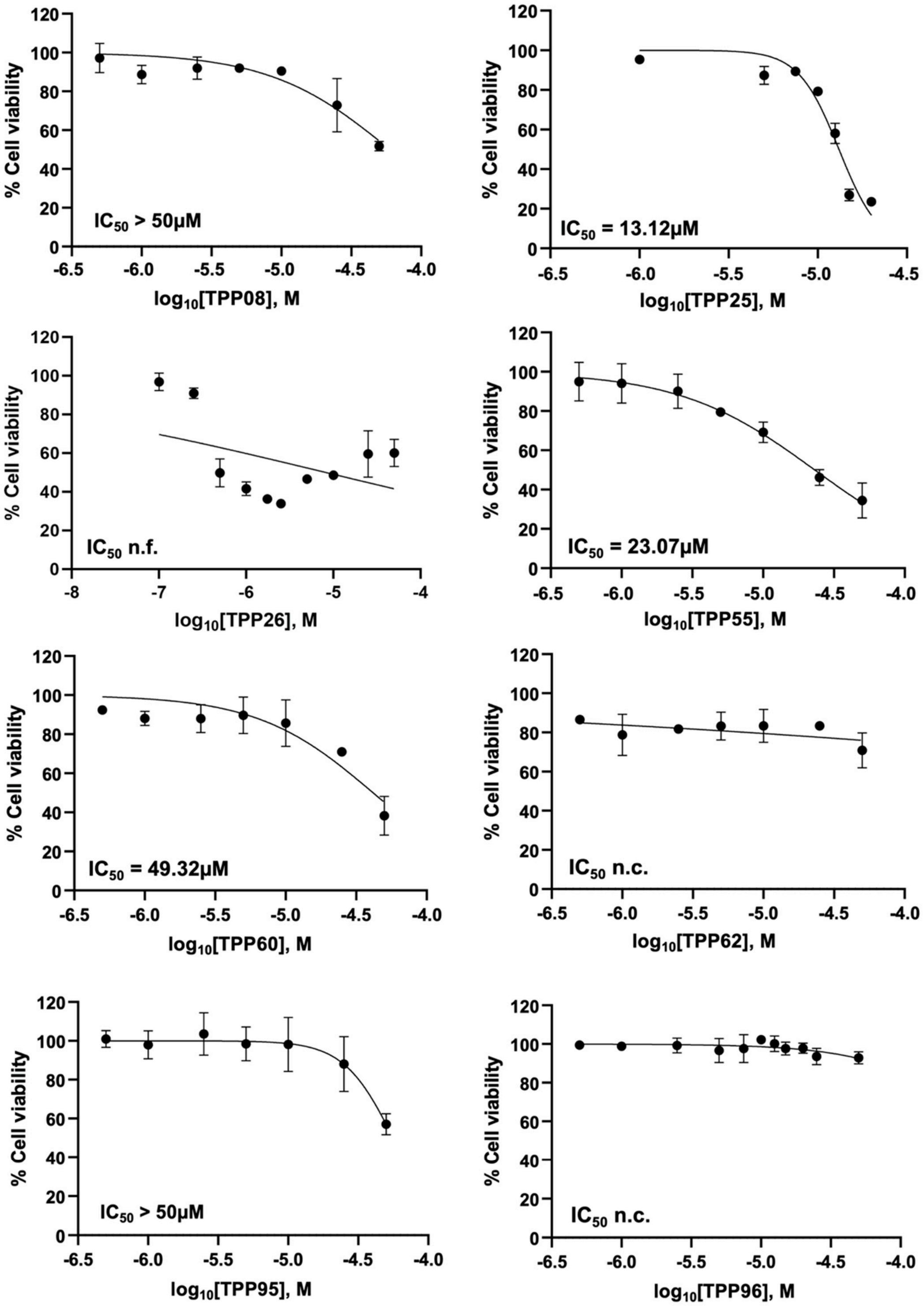

Figure S18. Cytotoxic effect of TPP compounds on U87 GBM cell line. Dose–response curves showing the percentage of cell viability (evaluated by MTT assay) as a function of the logarithmic concentration of the different compounds after 72 h of treatment. Concentrations on the x-axis are expressed as the base-10 logarithm of the molar concentration (log_{10}[TPP25], M). The respective IC_{50} values (half-maximal inhibitory concentration) are indicated for each compound. For curves where a standard IC_{50} could not be calculated: n.c. (not cytotoxic) indicates a lack of cytotoxicity within the tested concentration range; n.f. (not fittable) indicates an incomplete or non-sigmoidal cytotoxic profile where data could not be fitted to a standard sigmoidal model.

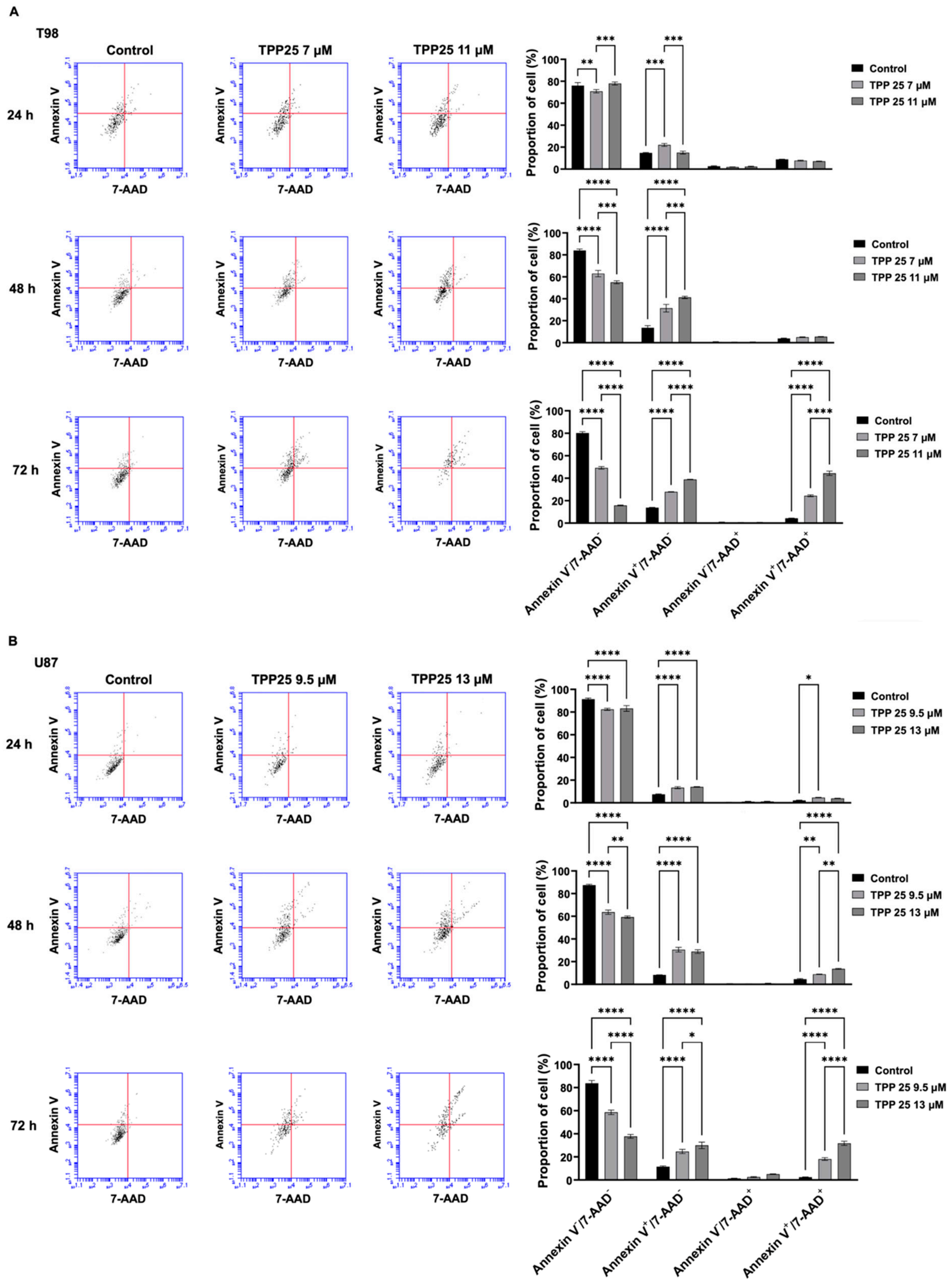

Figure S19. Annexin V/7-AAD-based assessment of TPP25-induced cell death in GBM cell lines. GBM cells (T98 and U87) were treated with TPP25 at the indicated concentrations for up to 72 h. Apoptosis was evaluated by flow cytometry following Annexin V-FITC/7-AAD staining. The percentages of viable cells (Annexin V$^-$/7-AAD$^-$), early apoptotic cells (Annexin V$^+$/7-AAD$^-$), late apoptotic cells (Annexin V$^+$/7-AAD$^+$), and necrotic cells (Annexin V$^-$/7-AAD$^+$) were quantified. Data are presented as mean ± SEM. Two-way ANOVA with Tukey's multiple comparison test was performed to analyze the individual and interactive effects of the two different treatment factors. *$p < 0.05$; **$p < 0.01$; ***$p < 0.001$; ****$p < 0.0001$.
